# A simplified in vitro disease-mimicking culture system can determine the angiogenic effect of medicines on vascular diseases

**DOI:** 10.1007/s10616-025-00736-4

**Published:** 2025-03-07

**Authors:** SongHo Moon, Yuzuru Ito

**Affiliations:** 1 Institute of Life and Environmental Sciences, University of Tsukuba, Tsukuba, Ibaraki Japan; 2 Life Science Development Department, CHIYODA Corporation, Yokohama, Kanagawa Japan; 3 National Institute of Advanced Industrial Science and Technology (AIST), Tsukuba, Ibaraki Japan

**Keywords:** Vascular diseases, Human umbilical vein endothelial cells, In vitro disease mimicking, Drug discovery model, Regenerative medicine

## Abstract

Many patients undergoing clinical regenerative treatments experience severe conditions arising from endothelial disruption. In chronic cardiac and perivascular diseases, deficiencies in vascular endothelial growth factor (VEGF), insulin-like growth factor (IGF), and heparin, which are essential for maintaining and activating endothelial cells, can lead to angiogenic dysregulation. Endothelial disruption caused by ischemic hypoxia and a deficiency in these factors is associated with many vascular diseases. However, their pathogenic processes remain unclear at the cellular level. Therefore, the present study aimed to develop a culture system that mimics the disease environment to test the effectiveness of drug candidates in restoring damaged blood vessels in chronic vascular diseases, including coronary artery disease and peripheral vascular disease. This study focused on VEGF, IGF, and heparin and developed a pseudo-disease culture system by pre-treating human umbilical vein endothelial cells (HUVECs) with a starvation medium (EGM-2™ medium lacking VEGF, IGF, and heparin) to examine the ability of HUVECs to form a traditional 2D vascular network. The results indicated that a deficiency in these proteins results in disruptions in tube morphogenesis. Moreover, the results suggested that dysregulation of the PI3K/AKT pathway plays a key role for in vascular disruption in HUVECs. The proposed pseudo-disease starvation system provides a simple way to visualize pathological disruptions to blood vessels and assess the efficacy of drugs for vascular regeneration.

**Supplementary Information:**

The online version contains supplementary material available at 10.1007/s10616-025-00736-4.

## Introduction

Regenerative medicine has predominantly been investigated in the context of chronic vascular diseases. Nevertheless, effective medicines for regenerating vessels affected by chronic vascular diseases remain unexplored. Understanding the pathogenic cellular reactions under disease conditions is essential for developing fundamental treatments against these vascular diseases. Therefore, a cell-based assay model, such as a drug screening system, is crucial to understanding cellular reactivity in vasculogenic regenerative medicines during pre-clinical trials. In particular, an in vitro screening system mimicking chronic vascular diseases, representing coronary artery disease (CAD) and peripheral vascular disease (PVD) clinical symptoms, is invaluable for studying therapeutic agents for vasculogenic recovery. However, research into CAD and PVD using in vivo vascular disease models is limited to knocking out specific factors, often leading to organ failure (Bellomo et al. 2000; Wu et al. 2012; Ock et al. 2016). Furthermore, confirming that the model is representative of CAD or PVD is challenging as it requires scanning predicted disease sites using angiography. Therefore, determining the chronological events leading to endothelial dysfunction and the ultimate failure of blood vessels to form vascular networks is essential.

Vascular diseases arise from endothelial disruption and dysfunction caused by ischemic stress, including ischemic stroke, heart diseases, diabetes, insulin resistance, chronic kidney failure, tumor growth, metastasis, venous thrombosis, and severe viral infectious diseases. In vascular diseases, at the cellular level, endothelial cells exhibit dysfunctional biology, suppressed immune response, and impaired metabolic synthesis (Rajendran et al. 2013). These effects may arise from ischemic abnormalities, including hypoxic conditions and essential nutrient deficiency caused by impaired blood flow. Such abnormalities are affected by dysregulated endogenic processes related to cellular proliferation, adhesion, migration, and metabolism, which are essential for maintaining and stabilizing the endothelium. In patients with acute circulatory diseases, these cellular functions are associated with high expression levels of vasculogenic genes. Notably, endothelial cells can remain dysfunctional even after the acute diagnostic symptoms have subsided. This decreased cellular function compared to that of unaffected sites, in patients with chronic diseases could be attributed to decreased blood vessel recovery (Karar and Maity 2011; Mamidi et al. 2012; Lu et al. 2011). Thus, chronic vascular diseases that develop following acute symptoms may be caused by a deficiency in factors essential for the long-term reconstruction of vessels. These factors may include insulin-like growth factor-1 (IGF-1), which is required for vessel generation throughout all processes, and vascular endothelial growth factor (VEGF), which is essential at disease sites for blood vessel formation (Amoli et al. 2012; Delafontaine et al. 2004; Peysselon and Ricard-Blum 2014; Higashi et al. 2019). However, the exact mechanisms underlying blood vessel formation at disease sites remain unknown, highlighting the importance of elucidating how blood vessels are reconstructed and stabilized at these sites.

We hypothesized that an endothelial cell-weakened culture system lacking VEGF, IGF, and heparin may reflect the diagnostic changes observed in vascular diseases leading to cellular dysfunction. Therefore, the present study aimed to investigate the effectiveness of drug candidates in restoring damaged blood vessels in chronic vascular diseases, including CAD and PVD, by developing a culture system that mimics the disease environment.

## Materials and methods

### Cell culture

For the vascular network and wound healing assays, normal human umbilical vein endothelial cells (HUVECs; C-2519AS; Lonza Bioscience, Walkersville, MD, USA) were seeded in 15 mL EGM-2™ medium (Lonza Bioscience) at 1.875 × 10^5^ cells/flask in T-75 flasks and grown in the same medium for 7 days. Cells were incubated at 37 °C in a 5% CO_2_ incubator. The culture medium was changed the day after thawing or seeding cells and then every other day thereafter. Cells were passaged or used for subsequent experiments when they reached approximately 70% confluency. All experiments in this study used cells at passage number P6.

### Establishment of a pseudo-CAD/PVD endothelial starvation mimic environment

A pseudo-CAD/PVD starvation medium was prepared using the contents of the EGM-2™ medium kit (Lonza Bioscience). VEGF, IGF, and heparin in the EGM-2™ kit were not added, whereas the other supplements were added at the recommended concentrations. After thawing, normal HUVECs were cultured in EGM-2™ in a T-75 flask for 19 h. The culture medium was discarded, and the cells were rinsed with 15 mL HEPES-BSS (Lonza Bioscience), followed by the addition of 15 mL of the pseudo-CAD/PVD medium. The cells were incubated for 6 days in the pseudo-CAD/PVD environment at 37 °C in a 5% CO_2_ incubator. The pseudo-CAD/PVD medium was changed the day after seeding and then every other day thereafter. Cells were passaged or used for subsequent experiments when they reached approximately 70% confluency. All cells were treated at passage numbers P5 to P6.

### 2-dimensional vascular network assay

An angiogenic assay on Matrigel™ was constructed as previously described (Arnaoutova and Kleinman 2010). Matrigel™ GFR (150 µL; BD Biosciences®, Franklin Lakes, NJ, USA) was injected into each well of a 48-well plate and gelated at 37 °C in a 5% CO_2_ incubator for 2 h. When HUVECs cultured in EGM-2™ or pseudo-CAD/PVD medium reached day 6 and a confluency of over 60%, they were detached by exposing them to 0.025% Trypsin/EDTA (Lonza Bioscience) for 3 min and collected using HEPES-BSS. The cells were then centrifuged at 200×*g* for 5 min at RT. Collected cells were diluted in their respective medium and then seeded at 1.6 × 10^5^ cells/mL in 250 µL of the diluted cell medium in each well of the prepared 48-well plate. Images of the blood vessel networks formed on the gelated Matrigel™ GFR were captured 19 h later using Bio Studio™ (Nikon, Tokyo, Japan). All experiments were performed in triplicate.

### Wound healing assay

The wound-healing ability of endothelial cells was assessed as previously described (Jonkman et al. 2014). HUVECs were seeded in 1 mL of EGM-2™ medium at 8 × 10^4^ cells/mL in each well of a 12-well plate. The cells were scratched using a P1000 pipette tip over an area of 2.57 × 10^6^ ± 1.8 × 10^5^ µm^2^. The cells were observed and imaged using Bio Studio™ and Bio Station™ (Nikon) at the start of the assay and after 4, 8, and 12 h. The scratched area was measured and standardized using ImageJ software (Fiji3.1.4). All experiments were performed in triplicate.

### Gene expression analysis

RNA was extracted from HUVECs using ISOGEN-LS (Nippon Gene, Tokyo, Japan) after pseudo-CAD/PVD treatment and at the 4 h and 19 h time points of the vascular network assay. Gene expression data were analyzed using GeneSpring™ 14.8 (Agilent Technologies, Santa Clara, CA, USA). All gene expressions were normalized using the 75th percentile-shift normalization method and filtered according to a fold change ≥ 2.0. The resulting 2818 filtered genes included 1262 upregulated and 1556 downregulated genes. After standardization of gene expression based on *p* ≤ 0.5, the final list comprised 1522 genes, including 620 upregulated and 902 downregulated genes. These 1522 genes were compared to data from the Kyoto Encyclopedia of Genes and Genomes (KEGG) pathway database and NCBI Gene database and analyzed according to cellular functions that could affect the ability of endothelial cells to form blood vessel networks and their wound-healing function. The genes were categorized into three types of angiogenic potential functions: blood vessel tubulogenic induction/inhibition, tumorigenic vessel induction/inhibition, and multiple tissue and neurovascular induction/inhibition. Based on this, a total of 89 genes were identified and their angiogenic effects were investigated, including 44 upregulated and 46 downregulated genes (one gene appeared twice after identification by different probes; Table 1, Online Resource 4). The literature on these genes was reviewed, and relationships with the PI3K/Akt signaling pathway were identified.

**Table 1:** Categorization of the cellular activities affected in HUVECs by pseudo-CAD/PVD treatment according to changes in 89 genes

Vasculogenic effect	Cellular activity changes under pseudo-CAD/PVD treatment	Direction of change in disease model
Angiogenesis	Endothelial Cell Junction Angiogenic Rearrangement Endothelial Migration Angiogenic Sprouting Chemokine Response	Up
	Endothelial Migration Angiogenic Rearrangement Cell-ECM Interaction Chemokine Response Angiogenic Sprouting Circular Canal Development Coronary Vascular Morphogenesis Endothelial Cell Junction Positive Angiogenic Regulation	Down
Multiple Tissue/Neuronal Vascular Induction	Cell Growth Cell Migration Cell–Cell Junction Axon Sprouting	Up
	Immune Response Insulin Resistance	Down
Tumorigenic Vascular Genesis	Tumor Growth Tumorigenic Vascular Invasion	Up
	Tumor Growth Tumorigenic Vascular Invasion Tumorigenic Signal Enhancement	Down

Negative vasculogenic effect	Cellular activity changes under pseudo-CAD/PVD treatment	Direction of change in disease model
Angiogenic Suppression	Inhibit Angiogenesis in Disease Negative Angiogenic Regulation Antiangiogenic Inflammation	Up
	Negative Angiogenic Regulation	Down
Tumorigenic Vascular Suppression	Tumor Suppression Tumor Vascular Inhibition Suppression of Epithelial Cell Migration	Up
	Tumor Vascular Inhibition Tumor Suppression	Down
Multiple Tissue/Neuronal Vascular Suppression	Tissue Involvement	Up

Genes falling under each cellular activity category are summarized in Online Resource 6

### Statistical analysis

To assess the healing effect in wound healing assays, the scratched areas were measured using ImageJ1.54i. Scratched area data were compared by calculating the mean value of triplicated samples for each culture condition. The measurements were analyzed via two-way analysis of variance (ANOVA) with GraphPad Prism9.5.1. Statistical significance was set at *p* < 0.05, indicated by an asterisk in the corresponding figure legends.

## Results

### Angiogenic starvation with a pseudo-CAD/PVD medium degrades the angiogenic ability of HUVECs

**Fig. 1:**
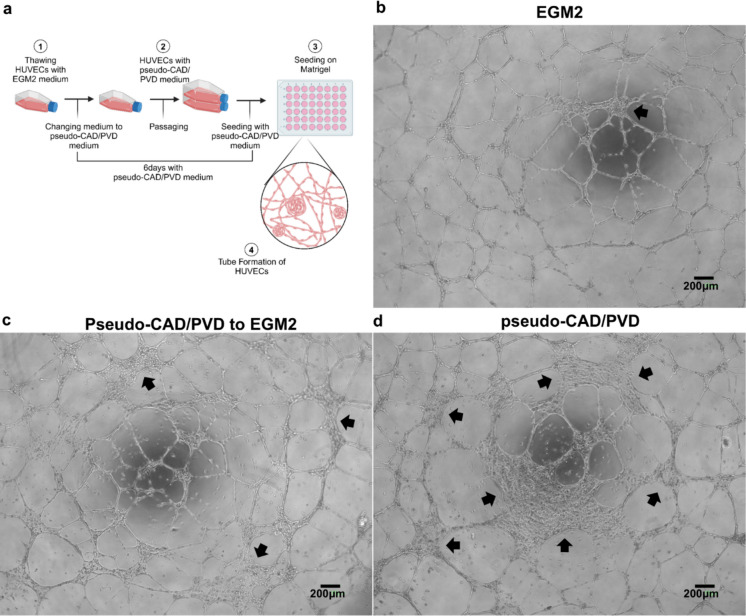
Effects of pseudo-coronary artery disease (CAD)/peripheral vascular disease (PVD) treatment in the tube formation assay. **a** Experimental scheme of the pseudo-CAD/PVD treatment. Morphology of 2-dimensional blood vessel networks formed by **b** control human umbilical vein endothelial cells (HUVECs), **c** CAD/PVD medium-treated HUVECs rescued by incubation in EGM2 medium, and **d** CAD/PVD medium-treated HUVECs. The arrows in each figure indicate abnormal HUVEC aggregation. The cell number was the same (4 × 10^4^ cells/well) in (**b**–**d**). All images were captured using Bio Studio™

The ability of endothelial cells to form a traditional 2D vascular network model on Matrigel™ was investigated to identify which endothelial cell functions required to construct blood vessel networks were affected in the pseudo-disease environment in vitro (Fig. 1a, Arnaoutova and Kleinman 2010). The co-reduction effect of VEGF, IGF, and heparin was compared by removing one hormone from the culture conditions. Notably, no considerable differences were observed when the groups under conditions lacking these hormones were compared to those under normal culture conditions (Online Resource 1). In contrast, after treating HUVECs with pseudo-CAD/PVD medium (EGM-2™ medium lacking VEGF, IGF, and heparin), HUVECs seeded on Matrigel™ demonstrated a decreased ability to form blood vessel networks. Specifically, the migratory ability of HUVECs in the pseudo-CAD/PVD medium group was substantially impaired compared to those under the control condition (Fig. 1b–d, arrows, Online Resource 2). Instead, large cell aggregates were observed between branches. Under the rescue condition, changing the medium to EGM2 in the tube formation assay after 6 days of pseudo-CAD/PVD treatment resulted in the formation of more apparent branches than those observed under the pseudo-CAD/PVD conditions; however, some aggregates remained visible. Despite the number of HUVECs being the same as that under control conditions, many of the treated cells did not migrate to positions necessary for sprouting new vessels and tightening endothelial cell-to-cell contacts in the vascular network. These results indicate that many treated HUVECs could not construct a blood vessel network as their migration ability was suppressed. However, it is important to note that the abnormal morphology made it challenging to measure the number of branch points and total length of blood vessels, two commonly used factors for evaluating the angiogenic potential of endothelial cells.

### Angiogenic degradation in the pseudo-CAD/PVD system is affected by endothelial migration

**Fig. 2:**
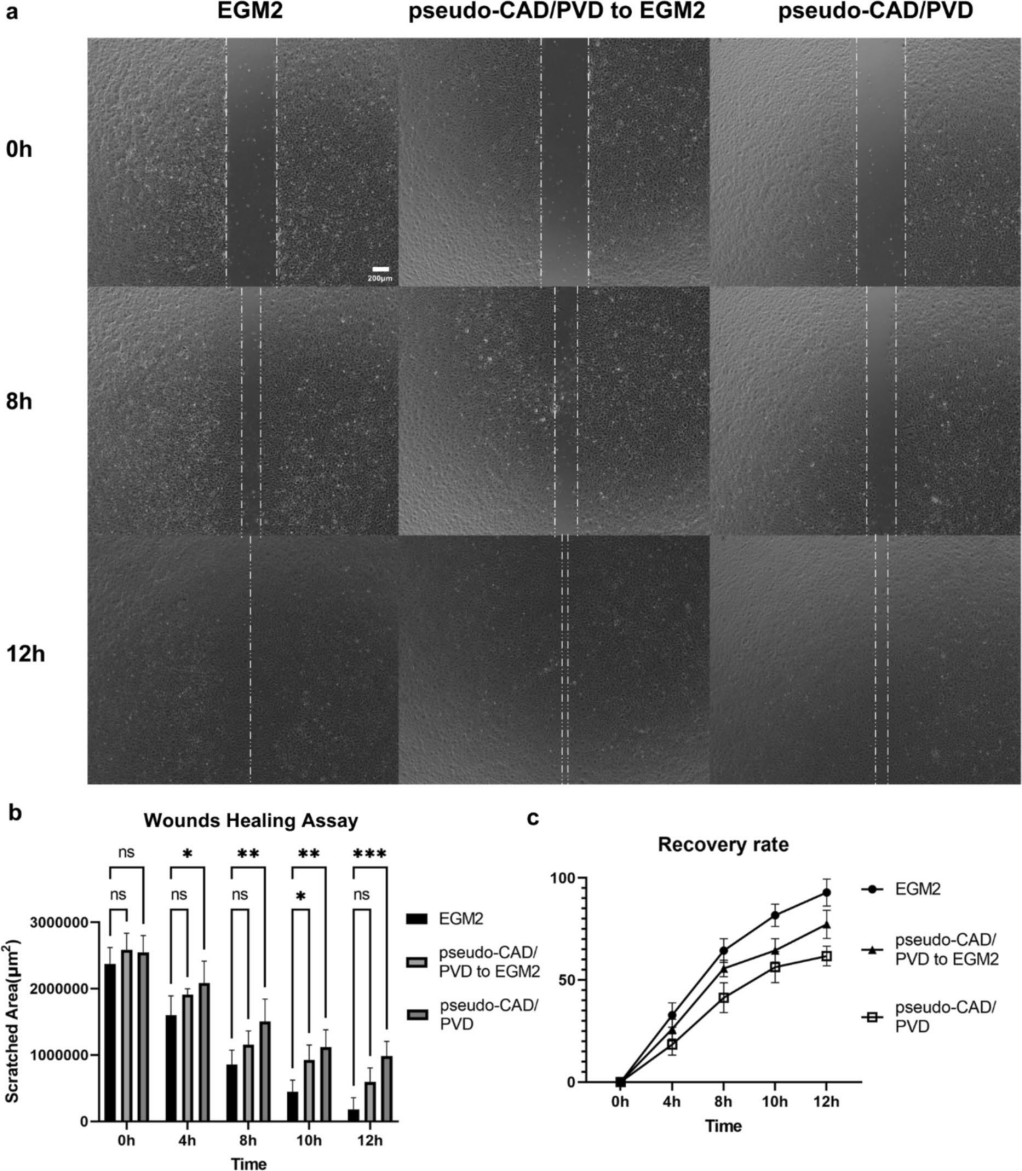
Effects of EGM2 and pseudo-CAD/PVD medium in the wound healing assay. **a** Changes in the detached cell area are indicated with a white dotted line, indicating the wound-healing ability of the cells after scratching them with a pipette tip. Healing ability differed among HUVECs pre-cultured and examined in EGM-2™ medium (EGM2), HUVECs pre-cultured and examined in pseudo-CAD/PVD medium (pseudo-CAD/PVD), and HUVECs pre-cultured in pseudo-CAD/PVD medium and examined in EGM-2™ medium (pseudo-CAD/PVD to EGM2). **b** Changes in the scratch area inflicted on cells using a pipette tip over time under the three conditions examined in **c**. The mean recovery rate of the scratch under the three conditions examined in **c**. Statistical data derived from results presented in (**b**, **c**) are shown in Online Resource 5. Error bars indicate standard deviation. *p < 0.05 in two-way ANOVA. All images were captured using Bio Studio™. (Color figure online)

Vascular diseases, including CAD or PVD, could differentially regulate endothelial gene expression, affecting endogenic cytokines required for immune responses, including inflammation and vascular induction (Williams et al. 2019; Hu et al. 2020). Therefore, a wound healing assay was performed using the same HUVEC groups as those in the vascular network assay to quantitatively examine the effect of the pseudo-CAD/PVD environment on cell migration. HUVECs that received in vitro pseudo-CAD/PVD treatment exhibited impaired wound-healing ability (Fig. 2a). After inducing cellular damage by physically scratching pseudo-CAD/PVD medium-treated and normal HUVECs with a pipette tip, the cells were cultured in EGM-2™ or pseudo-CAD/PVD medium, and their angiogenic potential was investigated (Fig. 2b and c). Notary, HUVECs cultured in regular EGM-2™ medium recovered from scratching even after long-term pseudo-CAD/PVD medium treatment. In contrast, cells cultured in pseudo-CAD/PVD medium after scratching exhibited impaired healing capability (Fig. 2b and c). Moreover, HUVECs treated with pseudo-CAD/PVD medium demonstrated approximately a 25% decrease in cell migration compared to normal and pseudo-CAD/PVD medium-treated HUVECs cultured in EGM-2™ medium. The effect of removing each growth factor individually on HUVEC migration was also investigated. However, no statistically significant differences were observed between the control to each condition (Online Resource 3–4).

### Angiogenic starvation with CAD/PVD medium alters the relative gene expression in HUVECs

Patients with vascular diseases exhibit significant changes in gene expression related to cellular proliferation, migration, and apoptosis in cells extracted from the diagnostic site and blood. In particular, patients with coronary and peripheral artery disease exhibit downregulated expression of genes associated with the proliferation and migration of endothelial and smooth muscle cells but upregulated expression of genes related to apoptosis (Sorrentino et al. 2020; Wu et al. 2021).

**Fig. 3:**
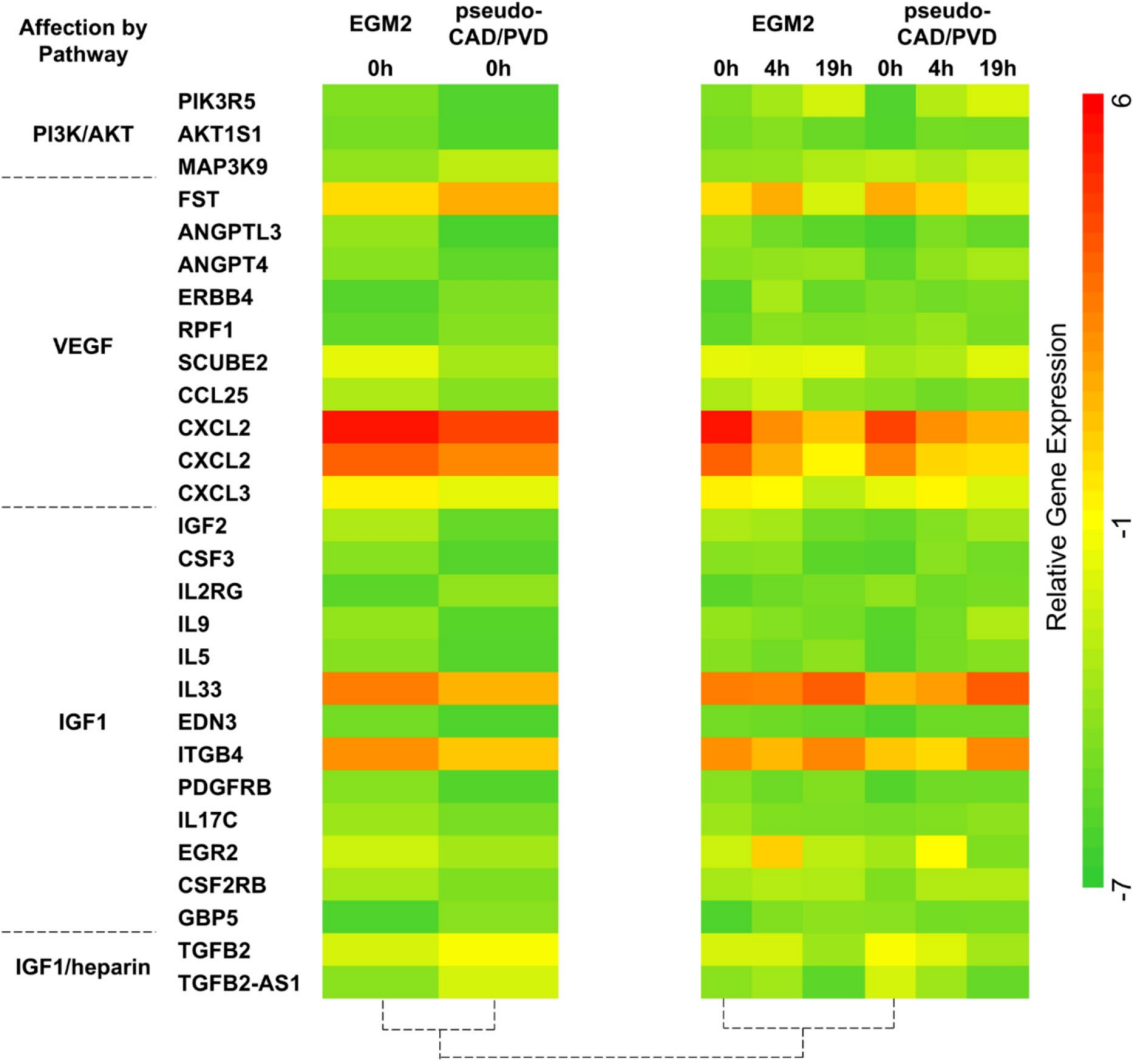
Heatmap of genes and pathways that directly or indirectly affect vascular genesis. Compared to normal HUVECs (EGM2), gene expression in pseudo-CAD/PVD-treated HUVECs (CAD) was changed after pseudo-CAD/PVD medium treatment and at 0 h in the 2-dimensional vascular network assay on Matrigel™. However, gene expression did not change at the 4 or 19 h time points in the vascular network assay. Gene expression was sorted according to the functional relationship of each gene with vascular genesis. Gene expression data were extracted from GeneSpring™ 14.8. VEGF, vascular endothelial growth factor; IGF-1, insulin-like growth factor-1. (Color figure online)

In this study, RNA was extracted from HUVECs before and after they formed 2-dimensional (2-D) vascular networks on Matrigel™ to evaluate the effect of pseudo-CAD/PVD medium treatment on gene expression. Changes in the regulation of gene expression were measured using a DNA microarray. A total of 1522 genes whose expression was significantly affected by pseudo-CAD/PVD medium treatment was investigated to determine the genes affecting the phenotype of HUVECs when forming 2-D vascular networks. A total of 89 genes were selected, among which 71 genes were found to play a role in generating normal or tumorigenic vessels, and 18 genes were found to inhibit vascular genesis (Fig. 3, Table 1, Online Resource 6). The expressions of these 89 genes involved in cell–cell adhesion, cell–extracellular matrix (ECM) adhesion, endothelial cell permeability, cell migration, tumorigenic vessel induction, tissue induction, and inflammatory reaction, were significantly altered at the start of vascular genesis on Matrigel™. Table 1 lists these genes according to their vasculogenic effect after pseudo-CAD/PVD medium treatment.

### Pseudo-CAD/PVD starvation disrupts genes related to vasculogenic migration in the PI3K pathway

Pathway analysis was conducted using GeneSpring™ 14.8 to identify common signaling pathways and determine the molecular details of the extracted genes. The results suggested that alterations in the expression of genes involved in phosphatidylinositol synthesis could affect the entire angiogenic process (Table 2, Online Resource 7). Notably, several of the 89 identified genes with functions related to vascular genesis in endothelial cells directly or indirectly affected the phosphatidylinositol 3-kinase/protein kinase B (PI3K/Akt) pathway.

**Table 2:** Pathway analysis of the 89 genes whose expression was altered by pseudo-CAD/PVD medium treatment

Pathway	EGM2 Vascular networking 0 h	Pseudo-CAD/PVD Vascular networking 0 h
D-myo-inositol(1,4,5,6)-tetrakisphosphate biosynthesis		
Inositol-polyphosphate kinase/phosphatase	− 3.23535	− 3.09952
Superpathway of D-myo-inositol(1,4,5)-trisphosphate metabolism		
Phosphatidyl inositol-4,5-bisphosphate5-phosphatase, A	− 3.62858	− 4.70854
Superpathway of inositol phosphate compounds		
Phosphatidylinositol-4,5,-Bisophosphate Phosphodiesterase Delta-4	− 6.13524	− 4.28159
Inositol-Polyphosphate kinase/Phosphatase	− 3.23535	− 3.09952
1-Phosphatidylinositol-4,5-Bisphosphate Phosphodiesterase Beta-1	− 0.45287	− 0.88368
Phosphatidylinositol-5-Phosphate4-Kinase	− 1.47582	− 1.22685
Phosphatidylinositol 4 Kinase Alpha	− 0.08661	− 0.64323
Phosphatidylinositol 3-Kinase, Classib, P110gamma/P101/	− 4.73091	− 6.06731
Phosphoinositide-3-Kinase Regulatory Subunit P101		
Phosphatidylinositol-4,5-Bisophosphate 5-Phosphatase, A	− 3.62858	− .4.70854

**Fig. 4:**
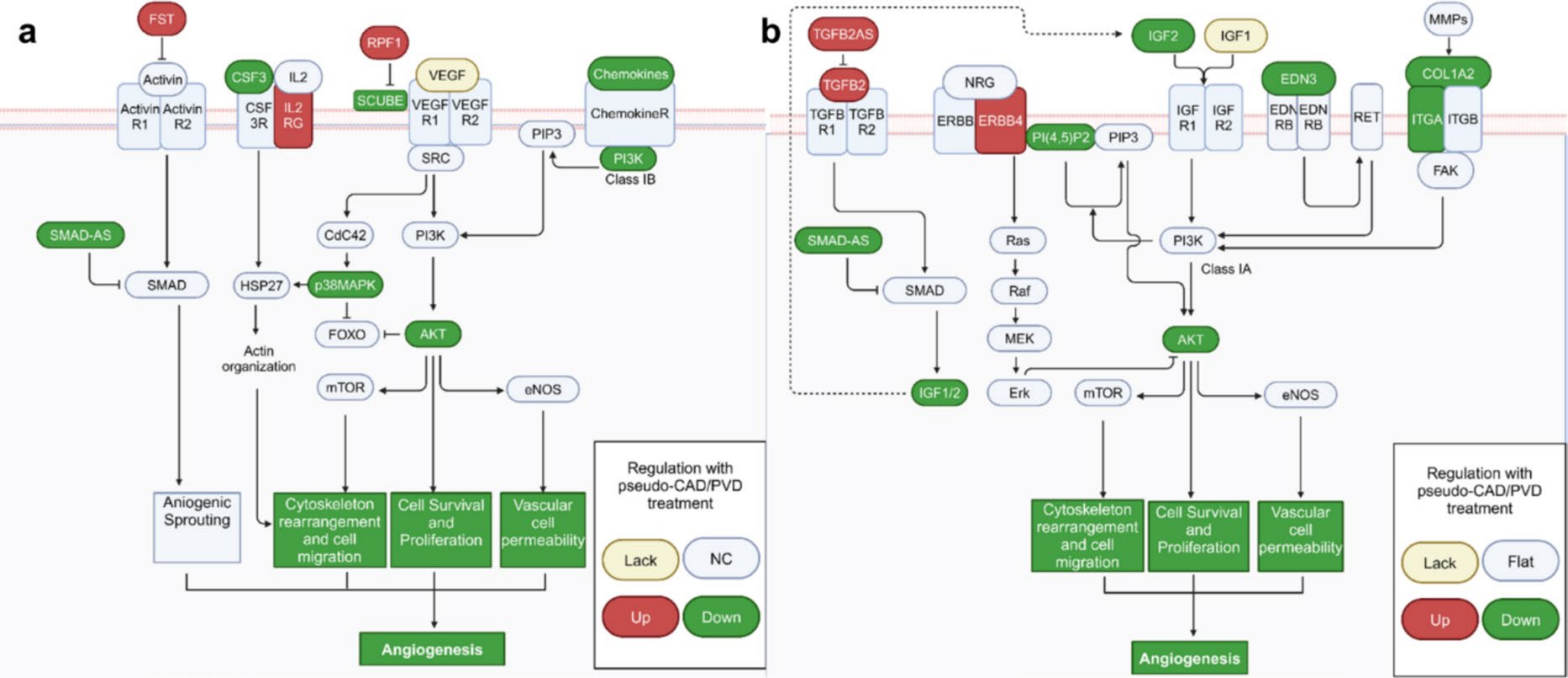
Involvement of PI3K/Akt signaling in the vasculogenic pathway. Genes related to vascular genesis that were upregulated and downregulated in the pseudo-CAD/PVD environment are indicated in red and green, respectively. **a** VEGF and **b** IGF deficiency affect various pathways through the PI3K/AKT pathway. Gene expression was only calculated after pseudo-CAD/PVD treatment. A summary showing all the affected factors is provided in Online Resource 7

Furthermore, the relative changes in the ability of endothelial cells to induce sprouting were screened vis pathway analysis using GeneSpring™ 14.8. Pathways were selected according to the smallest relative *p*-value (< 0.01) and categorized according to their relationship with the PI3K/Akt pathways. Specifically, the expression of *PIK3R5* (PI3K class 1B), *AKT1S1* (Peysselon and Ricard-Blum 2014; Lin et al. 2017), and *PI(4,5)P2*, encoding a precursor of PI3K class 1A, was considerably decreased, whereas that of *GBP5*, which suppresses PI3K class 1A synthesis (Liu et al. 2014), was increased (Fig. 4a). These findings suggest that CAD/PVD medium treatment directly inhibited the PI3K/AKT pathway and affected genes located upstream, including growth factors, chemokines, and those related to the ECM, which also indirectly inhibit the PI3K/Akt pathway. Moreover, the expression of other genes related to cell migration for vascular genesis, namely, *KRT16P3* (Ahmed et al. 2015), *POSTN* (Oka et al. 2007), *EGR3* (Liu et al. 2003; Yan et al. 2006), and *ANGPTL3* (Camenisch et al. 2002), which function with matrix metalloproteinases (MMP) in the ECM, was decreased, whereas that of *CMA1* (Orlowska-Baranowska et al. 2014; Meyer et al. 2017), which has a similar function, was increased.

In contrast, the expression of *GSN* (Huang et al. 2016), *Inc-CDH2-1* (Dorrell et al. 2002; Nalla et al. 2011), and *FST* (Li et al. 2014; Seachrist et al. 2017) was increased, enhancing activity in the PI3K/Akt pathway. However, no changes were observed in other genes associated with these genes. These findings suggest that pseudo-CAD/PVD medium treatment may not positively affect PI3K/Akt pathway activity. In particular, under this condition, it remained unclear whether the *FST* gene, which suppresses activin in the TGFB/activin pathway through SMAD (Icli et al. 2014), enhanced the PI3K pathway. This ambiguity regarding the effect of the upregulated expression of *FST* expression is because, despite an increase in *TGFB2* expression, the levels of its antisense gene, *TGFB2AS,* were also increased, whereas the expression of the end product of the same SMAD pathway and another antisense gene, *SMAD1-AS1*, decreased, suggesting TGFB/activin pathway suppression (Fig. 4b).

### Pseudo-CAD/PVD starvation disrupts vasculogenic genes related to the PI3K pathway

Changes in the expression of genes related to immune cytokines and ligands were observed, suggesting their potential impact on endothelial stability. While the expression levels of *CSF3* and its enhancer interleukin-5 (*IL-5*) were downregulated, both of which activate PI3K class 1A synthesis, the expression of *IL-2RG*, encoding a CSF3 ligand, was upregulated. Moreover, the expression of other ILs, such as *IL9* and *IL17C*, and the chemokine *CXCL3*, which positively affect the PI3K/Akt pathway, also decreased. Furthermore, the expression of the immune cytokine *IL33* and ligand *CD160*, which induces cytokine production, was also downregulated.

Notably, genes involved in tissue and tumor growth were also dysfunctionally regulated, indirectly affecting angiogenesis. Specifically, the expression of genes that directly suppress tissue development, namely *MIA2* (Xu et al. 2011), *EGOT* (Jin et al. 2017), and *NEURL4* (Cubillos-Rojas et al. 2017), was upregulated. Additionally, *CYP3A5*, which suppresses MMP2 and MMP9 activity—genes that degrade the interaction between the ECM and endothelial cells needed to initiate the formation of blood vessel networks (Jiang et al. 2015)—was upregulated. The expression of *GALNT14,* which activates MMPs in endothelial cells to induce sprouting, was downregulated. Moreover, the expression of *GREB1*, a gene involved in tumorigenic vascular genesis in tissues surrounding tumors, *GPM6B*, which suppresses tumorigenic vascular apoptosis, and *SERPINB7* and S*ERPINB10* (Valiente et al. 2014), which induce vascular expansion from normal tissues to tumors, were downregulated. In contrast, that of *GREB1L* (Hnatyszyn et al. 2010; Li et al. 2014), a gene that suppresses tumor growth, was upregulated.

Therefore, the activity of the PI3K/AKT pathway was directly inhibited to compare migration dysregulation under our pseudo-CAD/PVD condition. HUVECs were cultured for two passages under normal EGM-2 conditions and then subjected to tube formation assay with ZSTK474, a well-known potent inhibitor of the PI3K/AKT pathway. The results revealed that the constructed vessels exhibited apoptotic phenotypes in a concentration-dependent manner. However, their morphologies did not closely resemble those observed under our pseudo-CAD/PVD condition (Online Resource 8).

## Discussion

This study established a pseudo-disease culture system to mimic the morphology of diseased blood vessels and assess the effect on migration by treating endothelial cells with a pseudo-CAD/PVD medium, starving them of VEGF, IGF, and heparin. We first considered the activities of VEGF and other factors that influence angiogenic cascades. The effect of a deficiency in VEGF, IGF, and heparin in the culture condition was determined individually before assessing the effect of their combined deficiencies. Although VEGF plays a key role in vascular development, conditions of deficiency in VEGF, IGF, and heparin alone exhibited no significant differences including tube formation morphology compared to normal culture conditions. In contrast, pre-treating HUVECs with a pseudo-CAD/PVD medium altered the phenotype of the 2-D vascular network formed by HUVECs, possibly by disrupting vasculogenic tube formation and migration.

In the present study, solely assessing the morphology of the unhealthy vascular network was insufficient to quantify the change in vasculogenic cellular function; therefore, the changes in gene expression in endothelial cells after treatment were also assessed. Changes in the expression of several genes related to endothelial cell migration were observed, which may explain the deterioration in vasculogenic migration after pseudo-CAD/PVD medium treatment. We conducted a literature review of all 1522 genes using the Kyoto Encyclopedia of Genes and Genomes (KEGG) pathway database and published literature, focusing on their angiogenic, vasculogenic, tumorigenic, or neural morphogenic potential. Based on the Kyoto Encyclopedia of Genes and Genomes pathway database and published literature (Online Resource 6), 89 genes were found to be related to the PI3K/Akt signaling pathway through various signaling cascades. The cascades included PI3K/AKT, mTOR, VEGF, MAPK, chemokine, insulin, WNT, TGF-BETA, JAK-STAT, FOXO, TNF, HIGF1, ERBB, calcium, and phosphatidylinositol signaling pathways. Furthermore, the expression of genes related to the most important growth factor for vascular genesis, VEGF, was investigated. The expression levels of *SCUBE*, whose encoded protein forms a complex with VEGF and VEGFR2, were downregulated, whereas those of *RPF1*, which suppresses the formation of this complex (Rahimi 2009, 2012), were upregulated. This result suggests that RPF1—also known as NEDD4-1 and a member of the homologous to E6AP C-terminus family of E3 ligases—ubiquitinates target substrates attached to VEGFR2 through the ubiquitin pathway, leading to VEGFR2 activation (Murdaca et al. 2004). As RPF1 can also affect the non-canonical WNT pathway (Murdaca et al. 2004; Nielsen et al. 2019; Ding et al. 2013), the non-canonical WNT5A pathway may also inhibit VEGF activity. Additionally, WNT5A is suppressed by DKK1 (Smadja et al. 2010) and SCD5 (Sinner et al. 2012) in the canonical WNT pathway. In the present study, the expression of *DKK1* and SCD3 was increased in the pseudo-CAD/PVD group. In contrast, the relative expression of *ADTRP* (Patel et al. 2018) and *CCL25*, which induce cellular migration in the WNT canonical pathway, was decreased. Although the primary pathway involved in this disease-mimicking condition remains unclear, these findings suggest that both the canonical and non-canonical WNT pathways may have inhibited vasculogenic migration in our proposed disease-mimicking system.

This study had certain limitations. Although a pseudo-disease starvation model of critical factors was used to examine endogenous changes to sites affected by CAD and PVD, this system did not screen affected protein levels. Furthermore, the study only used endothelial cells and did not include any surrounding tissue, such as smooth muscle cells, a basement membrane, or blood flow, which could alter the effect of CAD/PVD medium treatment.

In conclusion, the proposed pseudo-disease environment may reflect the cellular dysfunction induced in various chronic vascular diseases. This pseudo-disease starvation system revealed disruptions in endothelial migration and sprouting, along with alterations in the expression of genes that regulate these functions. As many of these genes are associated with the PI3K/AKT pathway, these findings suggest that PI3K/AKT pathway dysregulation may play a key role in vascular disruption in disease conditions. Therefore, to further explore this, a PI3K/AKT inhibitor was used to determine the specific concentration that reflects the endothelial migration dysregulation observed under the pseudo-CAD/PVD condition during the tube formation assay. However, the results indicated that only abnormal, weak blood vessels or apoptotic phenotypes were constructed. Thus, the proposed pseudo-disease environment can serve as a useful model to test the efficacy of therapeutic agents in treating chronic vascular diseases. However, additional components, such as the surrounding tissue and blood flow, along with the quantification of PI3K/AKT activation level (e.g., determining AKT phosphorylation levels) must be investigated.

## Supplementary Information

Below is the link to the electronic supplementary material.

Supplementary file1 (DOCX 4901 KB)

Supplementary file2 (DOCX 2739 KB)

Supplementary file3 (DOCX 2372 KB)

Supplementary file4 (DOCX 37 KB)

Supplementary file5 (DOCX 30 KB)

Supplementary file6 (DOCX 50 KB)

Supplementary file7 (DOCX 124 KB)

Supplementary file8 (DOCX 1193 KB)

## Data Availability

The data that support the findings of this study are available from the corresponding author, Yuzuru Ito, upon reasonable request.
