# Supplementary material for: A simplified in vitro disease-mimicking culture system can determine the angiogenic effect of medicines on vascular diseases
Source: Cytotechnology. 2025 Mar 7;77(2):75. doi: 10.1007/s10616-025-00736-4 (PMC11889311; doi:10.1007/s10616-025-00736-4)
Supplement: Supplementary file 1 — Supplementary file1 (DOCX 4901 KB) [file 10616_2025_736_MOESM1_ESM.docx]

**Supplementary Information**

**A simplified *in vitro* disease-mimicking culture system can determine the angiogenic effect of medicines on vascular diseases**

SongHo Moon^1^, Yuzuru Ito^1,2,3*^

^1^Faculty of Life and Environmental Sciences, University of Tsukuba, Tsukuba, Ibaraki, Japan

^2^Life Science Development Department, CHIYODA Corporation, Yokohama, Kanagawa, Japan

^3^National Institute of Advanced Industrial Science and Technology (AIST), Tsukuba, Ibaraki, Japan

*Corresponding author

Yuzuru Ito

ORCID ID: 0000-0001-7923-865X

Email: [ito.yuzuru.fe@u.tsukuba.ac.jp](mailto:ito.yuzuru.fe@u.tsukuba.ac.jp)

**
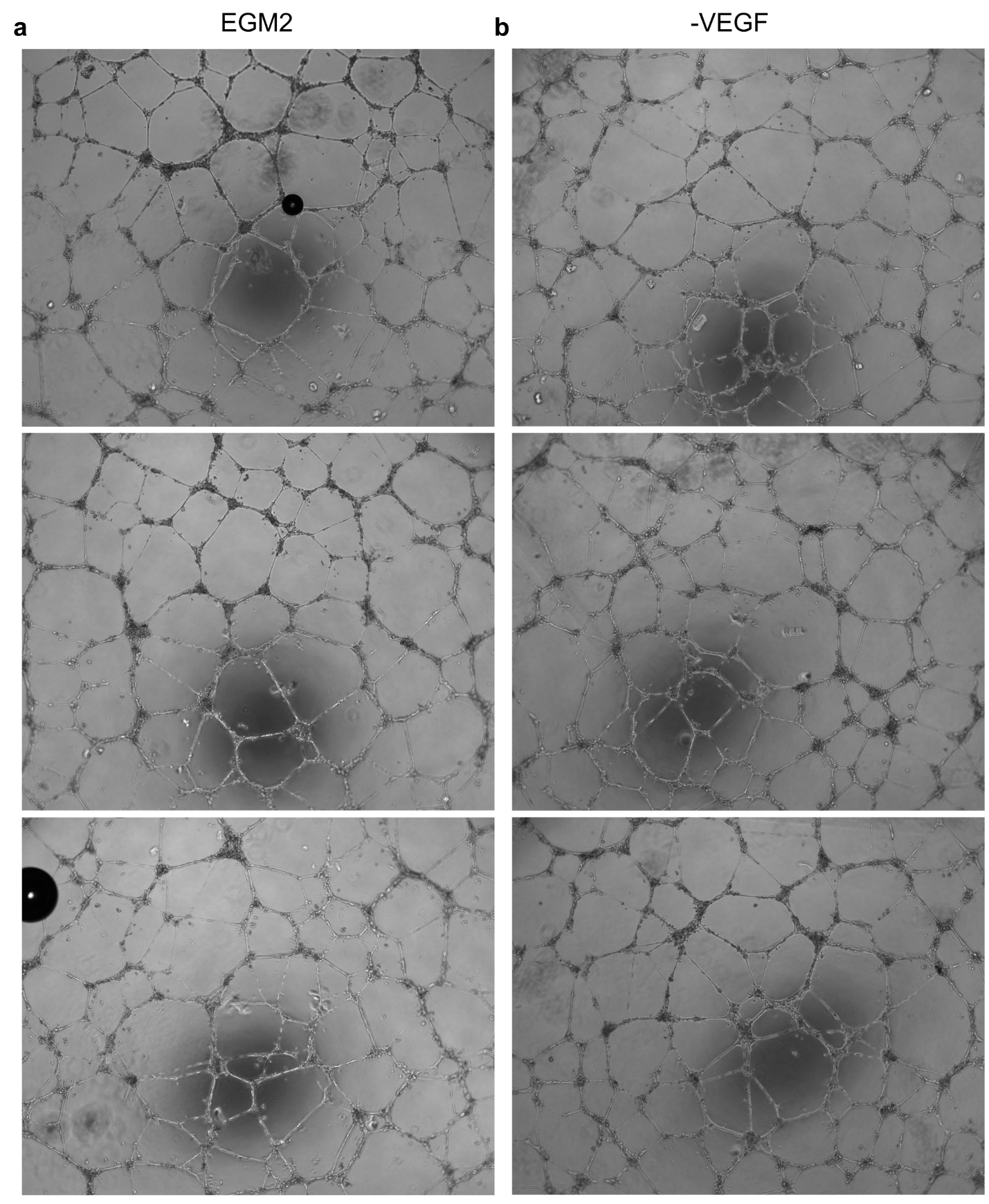

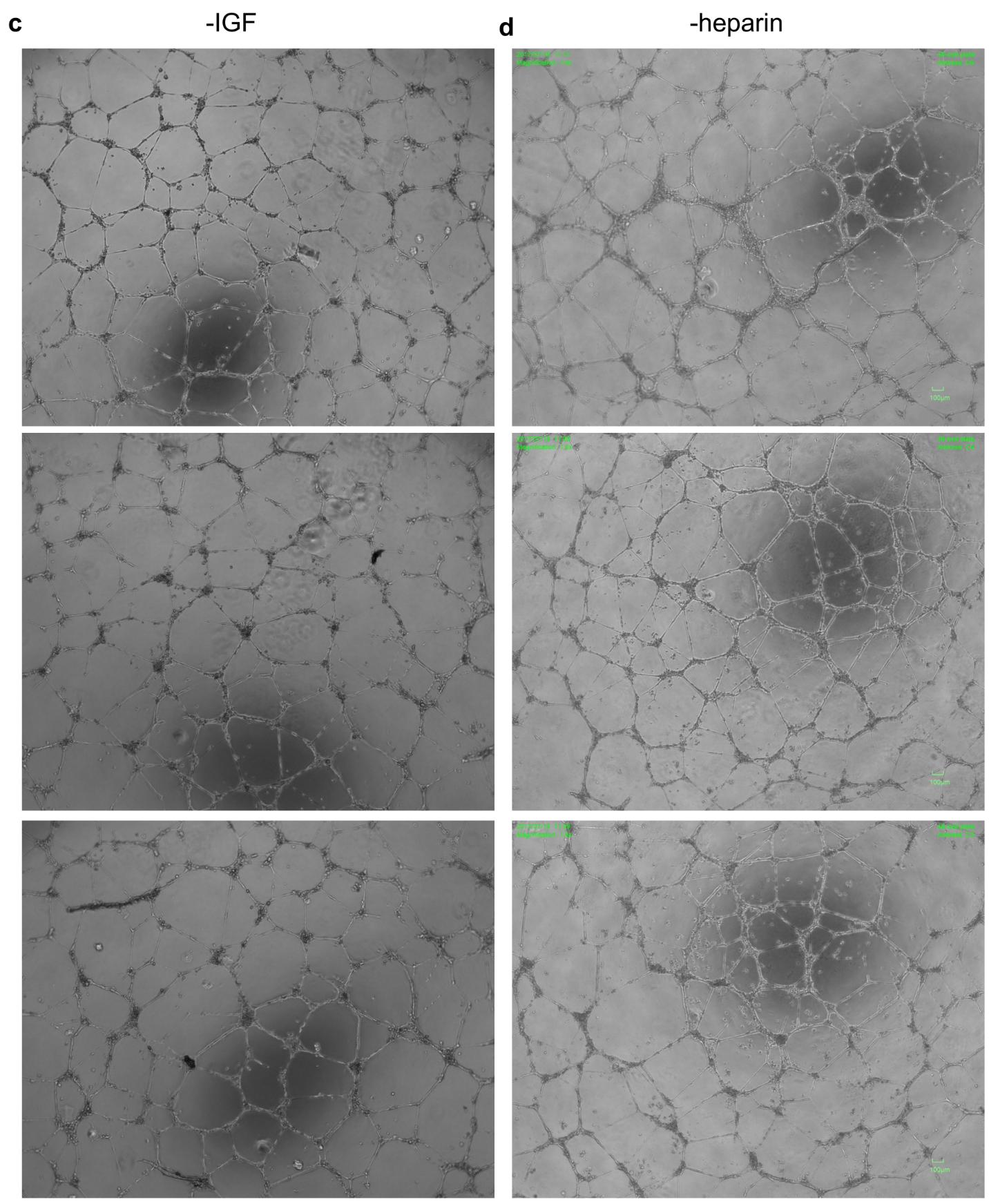
**

**Online Resource 1 The morphology of the 2-dimensional blood vessel network generated by cultured human umbilical vein endothelial cells (HUVECs) on Matrigel under the control condition (EGM2) and in the absence of each factor (vascular endothelial growth factor [-VEGF], insulin-like growth factor [-IGF] and -heparin).** Images in (a) and (b) were captured 19 h after seeding. The cell number was 40,000 cells/well. All images were captured on the same day (2022/07/15). The images in (c) and (d) were taken 19 h after seeding. The cell number was 40,000 cells/well. Images in (c) were taken on 2022/07/15, and images in (d) were taken on 2017/07/19
