# Supplementary material for: A simplified in vitro disease-mimicking culture system can determine the angiogenic effect of medicines on vascular diseases
Source: Cytotechnology. 2025 Mar 7;77(2):75. doi: 10.1007/s10616-025-00736-4 (PMC11889311; doi:10.1007/s10616-025-00736-4)
Supplement: Supplementary file 2 — Supplementary file2 (DOCX 2739 KB) [file 10616_2025_736_MOESM2_ESM.docx]

**Supplementary Information**

**A simplified *in vitro* disease-mimicking culture system can determine the angiogenic effect of medicines on vascular diseases**

SongHo Moon^1^, Yuzuru Ito^1,2,3*^

^1^Faculty of Life and Environmental Sciences, University of Tsukuba, Tsukuba, Ibaraki, Japan

^2^Life Science Development Department, CHIYODA Corporation, Yokohama, Kanagawa, Japan

^3^National Institute of Advanced Industrial Science and Technology (AIST), Tsukuba, Ibaraki, Japan

*Corresponding author

Yuzuru Ito

ORCID ID: 0000-0001-7923-865X

Email: [ito.yuzuru.fe@u.tsukuba.ac.jp](mailto:ito.yuzuru.fe@u.tsukuba.ac.jp)

**
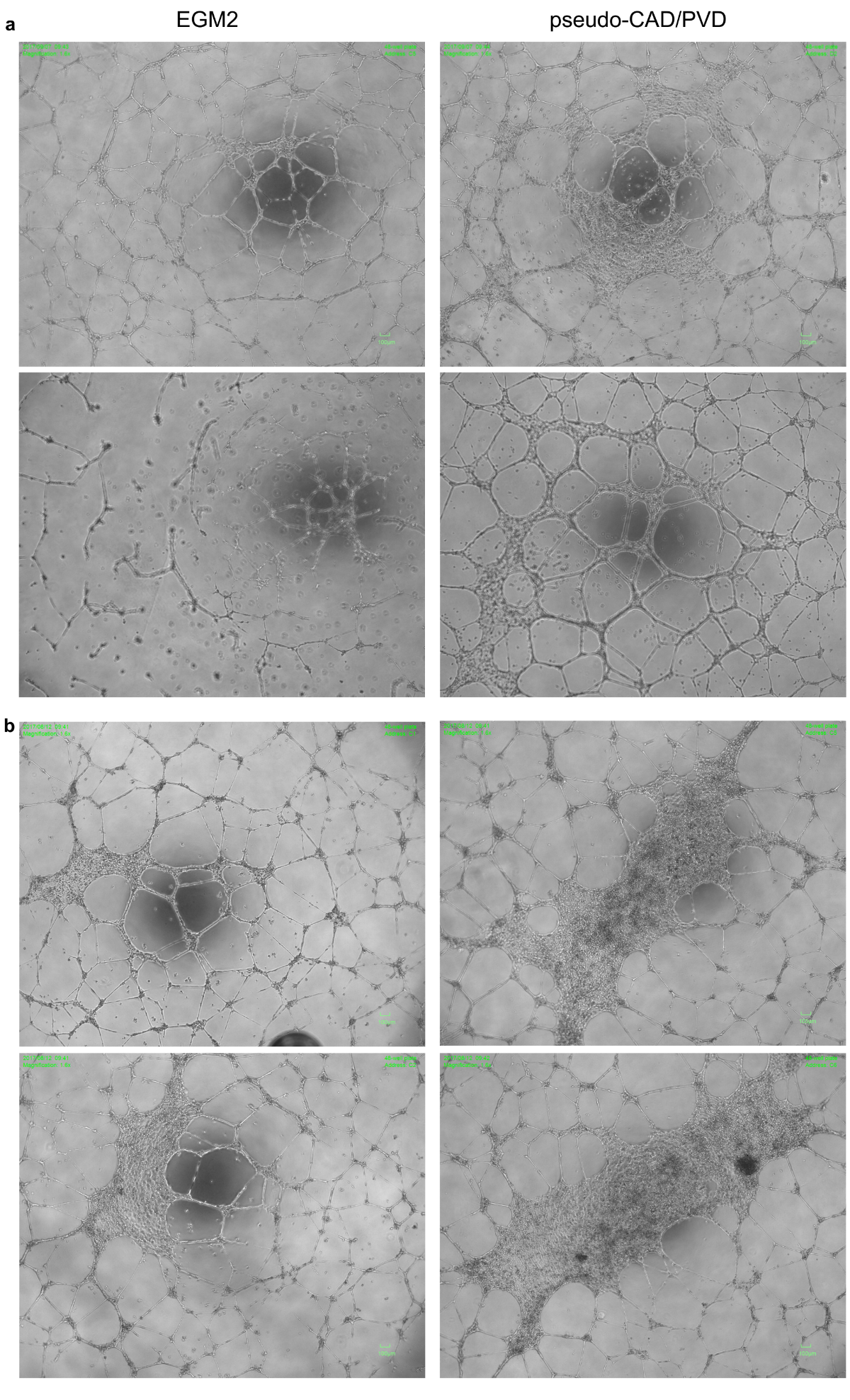
**

**Online Resource 2 Morphology of the 2-dimensional blood vessel network generated by cultured HUVECs on Matrigel under the control condition (EGM2) and coronary artery disease (CAD)/peripheral vascular diseases (PVD)treatment.** All images were taken 19 h after seeding. The cell number was 40,000 cells/well. All images were taken on the same day (a) 2017/09/07, (b) 2017/08/12
