# Supplementary material for: A simplified in vitro disease-mimicking culture system can determine the angiogenic effect of medicines on vascular diseases
Source: Cytotechnology. 2025 Mar 7;77(2):75. doi: 10.1007/s10616-025-00736-4 (PMC11889311; doi:10.1007/s10616-025-00736-4)
Supplement: Supplementary file 3 — Supplementary file3 (DOCX 2372 KB) [file 10616_2025_736_MOESM3_ESM.docx]

**Supplementary Information**

**A simplified *in vitro* disease-mimicking culture system can determine the angiogenic effect of medicines on vascular diseases**

SongHo Moon^1^, Yuzuru Ito^1,2,3*^

^1^Faculty of Life and Environmental Sciences, University of Tsukuba, Tsukuba, Ibaraki, Japan

^2^Life Science Development Department, CHIYODA Corporation, Yokohama, Kanagawa, Japan

^3^National Institute of Advanced Industrial Science and Technology (AIST), Tsukuba, Ibaraki, Japan

*Corresponding author

Yuzuru Ito

ORCID ID: 0000-0001-7923-865X

Email: [ito.yuzuru.fe@u.tsukuba.ac.jp](mailto:ito.yuzuru.fe@u.tsukuba.ac.jp)

**
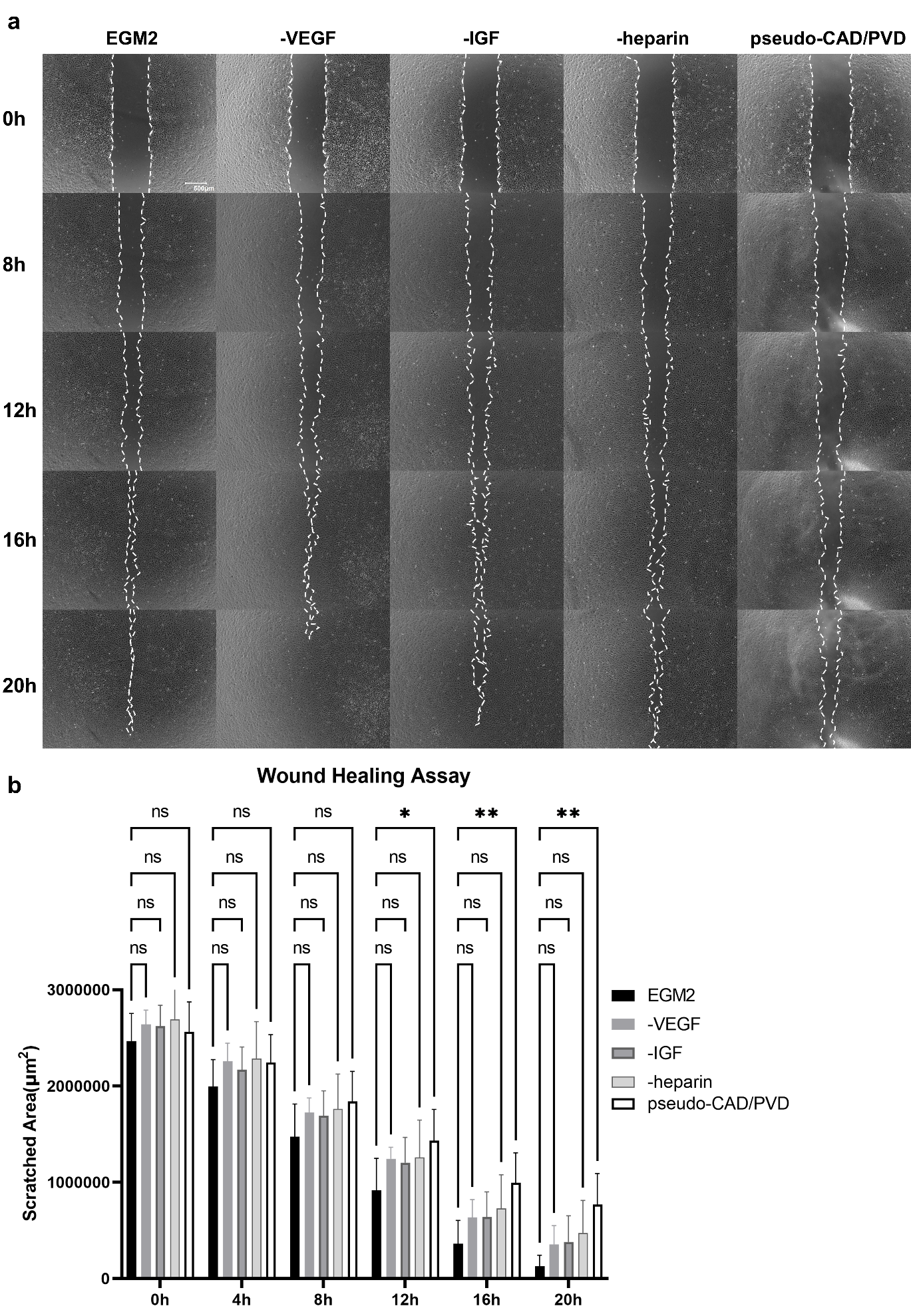
**

**Online Resource 3 Effect of individual growth factor reduced medium in wound healing assay.** The area of the scratched area were measured using ImageJ. (a) Changes in the detached cell area are indicated with a white line, representing the wound-healing ability of the cells after scratching them with a pipette tip. Healing ability differed among HUVECs pre-cultured and examined in an EGM-2™ medium (EGM2), VEGF-deficient EGM-2™ medium (-VEGF), IGF-deficient EGM-2™ medium (-IGF), heparin-deficient EGM-2™ medium (-heparin), and pseudo-CAD/PVD medium (pseudo-CAD/PVD). (b) Changes in the scratch area over time under the five examined conditions. Statistical data are derived from results presented in Online Resource 4. Error bars indicate standard deviation. **p* <0.05 using two-way ANOVA. All images were captured using Bio Studio™. ANOVA, analysis of variance; HUEVCs, human umbilical vein endothelial cells; VEGF, vascular endothelial growth factor; IGF, insulin-like growth factor; CAD, coronary artery disease; PVD, peripheral vascular disease; SE, standard error; Diff., difference; ns, not significant
