# Supplementary material for: A simplified in vitro disease-mimicking culture system can determine the angiogenic effect of medicines on vascular diseases
Source: Cytotechnology. 2025 Mar 7;77(2):75. doi: 10.1007/s10616-025-00736-4 (PMC11889311; doi:10.1007/s10616-025-00736-4)
Supplement: Supplementary file 4 — Supplementary file4 (DOCX 37 KB) [file 10616_2025_736_MOESM4_ESM.docx]

**Supplementary Information**

**A simplified *in vitro* disease-mimicking culture system can determine the angiogenic effect of medicines on vascular diseases**

SongHo Moon^1^, Yuzuru Ito^1,2,3*^

^1^Faculty of Life and Environmental Sciences, University of Tsukuba, Tsukuba, Ibaraki, Japan

^2^Life Science Development Department, CHIYODA Corporation, Yokohama, Kanagawa, Japan

^3^National Institute of Advanced Industrial Science and Technology (AIST), Tsukuba, Ibaraki, Japan

*Corresponding author

Yuzuru Ito

ORCID ID: 0000-0001-7923-865X

Email: [ito.yuzuru.fe@u.tsukuba.ac.jp](mailto:ito.yuzuru.fe@u.tsukuba.ac.jp)

| Table Analyzed | Data 1 |  |  |  |  |  |  |  |
| --- | --- | --- | --- | --- | --- | --- | --- | --- |
|  |  |  |  |  |  |  |  |  |
| Two-way RM ANOVA | Matching: Stacked | |  |  |  |  |  |  |
| Assume sphericity? | Yes |  |  |  |  |  |  |  |
| Alpha | 0.05 |  |  |  |  |  |  |  |
| Source of Variation | % of total variation | P value | P value summary | Significant? | |  |  |  |
| Row Factor x Column Factor | 0.7558 | <0.0001 | **** | Yes |  |  |  |  |
| Row Factor | 87.85 | <0.0001 | **** | Yes |  |  |  |  |
| Column Factor | 2.711 | 0.3364 | ns | No |  |  |  |  |
| Subject | 8.206 | <0.0001 | **** | Yes |  |  |  |  |
| ANOVA table | SS | DF | MS | F (DFn | DFd) | P value |  |  |
| Row Factor x Column Factor | 6.27E+11 | 20 | 3.13E+10 | F (20 | 75) = 5.929 | P<0.0001 |  |  |
| Row Factor | 7.29E+13 | 5 | 1.46E+13 | F (5 | 75) = 2757 | P<0.0001 |  |  |
| Column Factor | 2.25E+12 | 4 | 5.62E+11 | F (4 | 15) = 1.239 | P=0.3364 |  |  |
| Subject | 6.81E+12 | 15 | 4.54E+11 | F (15 | 75) = 85.85 | P<0.0001 |  |  |
| Residual | 3.96E+11 | 75 | 5.29E+09 |  |  |  |  |  |
| Data summary | |  |  |  |  |  |  |  |
| Number of columns (Column Factor) | 5 |  |  |  |  |  |  |  |
| Number of rows (Row Factor) | 6 |  |  |  |  |  |  |  |
| Number of subjects (Subject) | 20 |  |  |  |  |  |  |  |
| Number of missing values | 0 |  |  |  |  |  |  |  |
|  |  |  |  |  |  |  |  |  |
| Within each row, compare columns (simple effects within rows) | | | | | |  |  |  |
| Number of families | 6 |  |  |  |  |  |  |  |
| Number of comparisons per family | 4 |  |  |  |  |  |  |  |
| Alpha | 0.05 |  |  |  |  |  |  |  |
| Šídák's multiple comparisons test | Mean Diff. | 95.00% CI of diff. | Below threshold? | Summary | Adjusted P Value | |  |  |
|  |  |  |  |  |  |  |  |  |
| 0h |  |  |  |  |  |  |  |  |
| Control vs. -VEGF | -174526 | -682971 to 333919 | No | ns | 0.8572 |  |  |  |
| Control vs. -IGF | -155644 | -664089 to 352801 | No | ns | 0.9006 |  |  |  |
| Control vs. -heparin | -226421 | -734866 to 282025 | No | ns | 0.7013 |  |  |  |
| Control vs. CAD | -96619 | -605064 to 411827 | No | ns | 0.9813 |  |  |  |
|  |  |  |  |  |  |  |  |  |
| 4h |  |  |  |  |  |  |  |  |
| Control vs. -VEGF | -261436 | -769881 to 247009 | No | ns | 0.5792 |  |  |  |
| Control vs. -IGF | -174656 | -683101 to 333789 | No | ns | 0.8569 |  |  |  |
| Control vs. -heparin | -286663 | -795108 to 221782 | No | ns | 0.4909 |  |  |  |
| Control vs. CAD | -246333 | -754778 to 262112 | No | ns | 0.6324 |  |  |  |
|  |  |  |  |  |  |  |  |  |
| 8h |  |  |  |  |  |  |  |  |
| Control vs. -VEGF | -252696 | -761141 to 255749 | No | ns | 0.61 |  |  |  |
| Control vs. -IGF | -218379 | -726824 to 290066 | No | ns | 0.7281 |  |  |  |
| Control vs. -heparin | -286865 | -795310 to 221580 | No | ns | 0.4902 |  |  |  |
| Control vs. CAD | -366152 | -874597 to 142293 | No | ns | 0.2536 |  |  |  |
|  |  |  |  |  |  |  |  |  |
| 12h |  |  |  |  |  |  |  |  |
| Control vs. -VEGF | -326892 | -835337 to 181553 | No | ns | 0.3604 |  |  |  |
| Control vs. -IGF | -286831 | -795276 to 221614 | No | ns | 0.4903 |  |  |  |
| Control vs. -heparin | -339546 | -847991 to 168899 | No | ns | 0.3235 |  |  |  |
| Control vs. CAD | -515476 | -1023921 to -7031 | Yes | * | 0.0456 |  |  |  |
|  |  |  |  |  |  |  |  |  |
| 16h |  |  |  |  |  |  |  |  |
| Control vs. -VEGF | -273191 | -781636 to 235254 | No | ns | 0.5377 |  |  |  |
| Control vs. -IGF | -278018 | -786463 to 230428 | No | ns | 0.5209 |  |  |  |
| Control vs. -heparin | -365740 | -874185 to 142705 | No | ns | 0.2546 |  |  |  |
| Control vs. CAD | -632962 | -1141407 to -124516 | Yes | ** | 0.0085 |  |  |  |
|  |  |  |  |  |  |  |  |  |
| 20h |  |  |  |  |  |  |  |  |
| Control vs. -VEGF | -227451 | -735896 to 280994 | No | ns | 0.6978 |  |  |  |
| Control vs. -IGF | -254358 | -762803 to 254087 | No | ns | 0.6042 |  |  |  |
| Control vs. -heparin | -345913 | -854358 to 162532 | No | ns | 0.3058 |  |  |  |
| Control vs. CAD | -640671 | -1149117 to -132226 | Yes | ** | 0.0075 |  |  |  |
|  |  |  |  |  |  |  |  |  |
|  |  |  |  |  |  |  |  |  |
| Test details | Mean 1 | Mean 2 | Mean Diff. | SE of diff. | N1 | N2 | t | DF |
|  |  |  |  |  |  |  |  |  |
| 0h |  |  |  |  |  |  |  |  |
| Control vs. -VEGF | 2466475 | 2641001 | -174526 | 200039 | 4 | 4 | 0.8725 | 90 |
| Control vs. -IGF | 2466475 | 2622119 | -155644 | 200039 | 4 | 4 | 0.7781 | 90 |
| Control vs. -heparin | 2466475 | 2692895 | -226421 | 200039 | 4 | 4 | 1.132 | 90 |
| Control vs. CAD | 2466475 | 2563093 | -96619 | 200039 | 4 | 4 | 0.483 | 90 |
|  |  |  |  |  |  |  |  |  |
| 4h |  |  |  |  |  |  |  |  |
| Control vs. -VEGF | 1996899 | 2258335 | -261436 | 200039 | 4 | 4 | 1.307 | 90 |
| Control vs. -IGF | 1996899 | 2171555 | -174656 | 200039 | 4 | 4 | 0.8731 | 90 |
| Control vs. -heparin | 1996899 | 2283562 | -286663 | 200039 | 4 | 4 | 1.433 | 90 |
| Control vs. CAD | 1996899 | 2243233 | -246333 | 200039 | 4 | 4 | 1.231 | 90 |
|  |  |  |  |  |  |  |  |  |
| 8h |  |  |  |  |  |  |  |  |
| Control vs. -VEGF | 1474595 | 1727290 | -252696 | 200039 | 4 | 4 | 1.263 | 90 |
| Control vs. -IGF | 1474595 | 1692974 | -218379 | 200039 | 4 | 4 | 1.092 | 90 |
| Control vs. -heparin | 1474595 | 1761460 | -286865 | 200039 | 4 | 4 | 1.434 | 90 |
| Control vs. CAD | 1474595 | 1840747 | -366152 | 200039 | 4 | 4 | 1.83 | 90 |
|  |  |  |  |  |  |  |  |  |
| 12h |  |  |  |  |  |  |  |  |
| Control vs. -VEGF | 918203 | 1245096 | -326892 | 200039 | 4 | 4 | 1.634 | 90 |
| Control vs. -IGF | 918203 | 1205034 | -286831 | 200039 | 4 | 4 | 1.434 | 90 |
| Control vs. -heparin | 918203 | 1257749 | -339546 | 200039 | 4 | 4 | 1.697 | 90 |
| Control vs. CAD | 918203 | 1433679 | -515476 | 200039 | 4 | 4 | 2.577 | 90 |
|  |  |  |  |  |  |  |  |  |
| 16h |  |  |  |  |  |  |  |  |
| Control vs. -VEGF | 362053 | 635244 | -273191 | 200039 | 4 | 4 | 1.366 | 90 |
| Control vs. -IGF | 362053 | 640071 | -278018 | 200039 | 4 | 4 | 1.39 | 90 |
| Control vs. -heparin | 362053 | 727793 | -365740 | 200039 | 4 | 4 | 1.828 | 90 |
| Control vs. CAD | 362053 | 995015 | -632962 | 200039 | 4 | 4 | 3.164 | 90 |
|  |  |  |  |  |  |  |  |  |
| 20h |  |  |  |  |  |  |  |  |
| Control vs. -VEGF | 128716 | 356167 | -227451 | 200039 | 4 | 4 | 1.137 | 90 |
| Control vs. -IGF | 128716 | 383074 | -254358 | 200039 | 4 | 4 | 1.272 | 90 |
| Control vs. -heparin | 128716 | 474629 | -345913 | 200039 | 4 | 4 | 1.729 | 90 |
| Control vs. CAD | 128716 | 769387 | -640671 | 200039 | 4 | 4 | 3.203 | 90 |

**Online Resource 4 Calculation of the effect of individual growth factor reduced medium in wound healing assay**

The area of the scratched area were measured using imageJ. **p* <0.05, ***p* <0.01 in two-way ANOVA. All images were captured using Bio Studio™. ANOVA, analysis of variance; HUEVCs, human umbilical vein endothelial cells; VEGF, vascular endothelial growth factor; IGF, insulin-like growth factor; CAD, coronary artery disease; PVD, peripheral vascular disease; SE, standard error; Diff., difference; ns, not significant
