# Supplementary material for: A simplified in vitro disease-mimicking culture system can determine the angiogenic effect of medicines on vascular diseases
Source: Cytotechnology. 2025 Mar 7;77(2):75. doi: 10.1007/s10616-025-00736-4 (PMC11889311; doi:10.1007/s10616-025-00736-4)
Supplement: Supplementary file 5 — Supplementary file5 (DOCX 30 KB) [file 10616_2025_736_MOESM5_ESM.docx]

**Supplementary Information**

**A simplified *in vitro* disease-mimicking culture system can determine the angiogenic effect of medicines on vascular diseases**

SongHo Moon^1^, Yuzuru Ito^1,2,3*^

^1^Faculty of Life and Environmental Sciences, University of Tsukuba, Tsukuba, Ibaraki, Japan

^2^Life Science Development Department, CHIYODA Corporation, Yokohama, Kanagawa, Japan

^3^National Institute of Advanced Industrial Science and Technology (AIST), Tsukuba, Ibaraki, Japan

*Corresponding author

Yuzuru Ito

ORCID ID: 0000-0001-7923-865X

Email: [ito.yuzuru.fe@u.tsukuba.ac.jp](mailto:ito.yuzuru.fe@u.tsukuba.ac.jp)

| Area Measurement of Scratch Assay(μ㎡) | | | |
| --- | --- | --- | --- |
| Time | EGM2 | pseudo- CAD/PVD  to EGM2 | pseudo- CAD/PVD |
| 0h | 2358262 | 2313128.217 | 2832590.348 |
| 0h | 2629068 | 2667491.262 | 2346978.874 |
| 0h | 2132591 | 2787037.413 | 2468475.405 |
| Mean 0h | 2373307 | 2589218.964 | 2549348.209 |
| Stdev 0h | 202964.6 | 201233.5227 | 206332.957 |
| 4h | 1669966 | 1839219.022 | 2468288.08 |
| 4h | 1850503 | 1881609.476 | 1896275.891 |
| 4h | 1286325 | 2008472.306 | 1896617.482 |
| Mean 4h | 1602264 | 1909766.935 | 2087060.484 |
| Stdev 4h | 235247 | 71908.74029 | 269568.6541 |
| 8h | 880117.1 | 936534.839 | 1896551.368 |
| 8h | 1060654 | 1173489.437 | 1312781.805 |
| 8h | 620595.4 | 1354026.274 | 1312858.938 |
| Mean 8h | 853788.8 | 1154683.517 | 1507397.37 |
| Stdev 8h | 180615.2 | 170958.1266 | 275173.4325 |
| 10h | 417491.4 | 733430.898 | 1312980.148 |
| 10h | 631878.9 | 873792.118 | 825032.627 |
| 10h | 282088.8 | 1173489.437 | 1225631.79 |
| Mean 10h | 443819.7 | 926904.151 | 1121214.855 |
| Stdev 10h | 144009.6 | 183536.6366 | 212446.6491 |
| 12h | 169088 | 508729.533 | 1225797.076 |
| 12h | 361073.7 | 434174.343 | 790091.08 |
| 12h | 10391.01 | 834982.869 | 933581.722 |
| Mean 12h | 180184.2 | 592628.915 | 983156.626 |
| Stdev 12h | 143380.4 | 174052.0963 | 181297.5125 |

| Table Analyzed | Scratched area | |  |  |  |  |
| --- | --- | --- | --- | --- | --- | --- |
|  |  |  |  |  |  |  |
| Two-way ANOVA | Ordinary |  |  |  |  |  |
| Alpha | 0.05 |  |  |  |  |  |
|  |  |  |  |  |  |  |
| Source of Variation | % of total variation | P value | P value summary | Significant? | |  |
| Interaction | 1.474 | 0.5648 | ns | No |  |  |
| Row Factor | 83.15 | <0.0001 | **** | Yes |  |  |
| Column Factor | 8.9 | <0.0001 | **** | Yes |  |  |
|  |  |  |  |  |  |  |
| ANOVA table | SS | DF | MS | F (DFn | DFd) | P value |
| Interaction | 3.95E+11 | 8 | 4.94E+10 | F (8 | 30) = 0.8535 | P=0.5648 |
| Row Factor | 2.23E+13 | 4 | 5.57E+12 | F (4 | 30) = 96.33 | P<0.0001 |
| Column Factor | 2.38E+12 | 2 | 1.19E+12 | F (2 | 30) = 20.62 | P<0.0001 |
| Residual | 1.73E+12 | 30 | 5.78E+10 |  |  |  |
|  |  |  |  |  |  |  |
| Data summary | |  |  |  |  |  |
| Number of columns (Column Factor) | 3 |  |  |  |  |  |
| Number of rows (Row Factor) | 5 |  |  |  |  |  |
| Number of values | 45 |  |  |  |  |  |

| Dunnett's multiple comparisons test | Mean Diff. | 95.00% CI of diff. | Below threshold | Summary | Adjusted P Value |
| --- | --- | --- | --- | --- | --- |
| 0h |  |  |  |  |  |
| vs. pseudo-CAD/PVD to EGM2 | -215912 | -671610 to 239786 | No | ns | 0.4481 |
| vs. pseudo-CAD/PVD | -176041 | -631739 to 279657 | No | ns | 0.5774 |
| 4h |  |  |  |  |  |
| vs. pseudo-CAD/PVD to EGM2 | -307502 | -763200 to 148196 | No | ns | 0.2192 |
| vs. pseudo-CAD/PVD | -484796 | -940494 to -29098 | Yes | * | 0.036 |
| 8h |  |  |  |  |  |
| vs. pseudo-CAD/PVD to EGM2 | -300894 | -756592 to 154803 | No | ns | 0.2321 |
| vs. pseudo-CAD/PVD | -653609 | -1109306 to -197911 | Yes | ** | 0.0044 |
| 10h |  |  |  |  |  |
| vs. pseudo-CAD/PVD to EGM2 | -483084 | -938782 to -27386 | Yes | * | 0.0367 |
| vs. pseudo-CAD/PVD | -677395 | -1133093 to -221697 | Yes | ** | 0.0032 |
| 12h |  |  |  |  |  |
| vs. pseudo-CAD/PVD to EGM2 | -412445 | -868143 to 43253 | No | ns | 0.0797 |
| vs. pseudo-CAD/PVD | -802972 | -1258670 to -347275 | Yes | *** | 0.0006 |

| Test details | Mean 1  (EGM2) | Mean 2 | Mean Diff. | SE of diff. | N1 | N2 | q | DF |
| --- | --- | --- | --- | --- | --- | --- | --- | --- |
| 0h |  |  |  |  |  |  |  |  |
| vs. pseudo-CAD/PVD to EGM2 | 2373307 | 2589219 | -215912 | 196348 | 3 | 3 | 1.1 | 30 |
| vs. pseudo-CAD/PVD | 2373307 | 2549348 | -176041 | 196348 | 3 | 3 | 0.8966 | 30 |
| 4h |  |  |  |  |  |  |  |  |
| vs. pseudo-CAD/PVD to EGM2 | 1602265 | 1909767 | -307502 | 196348 | 3 | 3 | 1.566 | 30 |
| vs. pseudo-CAD/PVD | 1602265 | 2087060 | -484796 | 196348 | 3 | 3 | 2.469 | 30 |
| 8h |  |  |  |  |  |  |  |  |
| vs. pseudo-CAD/PVD to EGM2 | 853789 | 1154683 | -300894 | 196348 | 3 | 3 | 1.532 | 30 |
| vs. pseudo-CAD/PVD | 853789 | 1507397 | -653609 | 196348 | 3 | 3 | 3.329 | 30 |
| 10h |  |  |  |  |  |  |  |  |
| vs. pseudo-CAD/PVD to EGM2 | 443820 | 926904 | -483084 | 196348 | 3 | 3 | 2.46 | 30 |
| vs. pseudo-CAD/PVD | 443820 | 1121215 | -677395 | 196348 | 3 | 3 | 3.45 | 30 |
| 12h |  |  |  |  |  |  |  |  |
| vs. pseudo-CAD/PVD to EGM2 | 180184 | 592629 | -412445 | 196348 | 3 | 3 | 2.101 | 30 |
| vs. pseudo-CAD/PVD | 180184 | 983157 | -802972 | 196348 | 3 | 3 | 4.09 | 30 |

**Online Resource 5 Recovery rate calculations were conducted in the wound healing assay using ImageJ software.** The area of the scratched area were measured.
