# Supplementary material for: A simplified in vitro disease-mimicking culture system can determine the angiogenic effect of medicines on vascular diseases
Source: Cytotechnology. 2025 Mar 7;77(2):75. doi: 10.1007/s10616-025-00736-4 (PMC11889311; doi:10.1007/s10616-025-00736-4)
Supplement: Supplementary file 6 — Supplementary file6 (DOCX 50 KB) [file 10616_2025_736_MOESM6_ESM.docx]

**Supplementary Information**

**A simplified *in vitro* disease-mimicking culture system can determine the angiogenic effect of medicines on vascular diseases**

SongHo Moon^1^, Yuzuru Ito^1,2,3*^

^1^Faculty of Life and Environmental Sciences, University of Tsukuba, Tsukuba, Ibaraki, Japan

^2^Life Science Development Department, CHIYODA Corporation, Yokohama, Kanagawa, Japan

^3^National Institute of Advanced Industrial Science and Technology (AIST), Tsukuba, Ibaraki, Japan

*Corresponding author

Yuzuru Ito

ORCID ID: 0000-0001-7923-865X

Email: [ito.yuzuru.fe@u.tsukuba.ac.jp](mailto:ito.yuzuru.fe@u.tsukuba.ac.jp)

**Online Resource 6 Categorization of 89 genes, of which (a) 71 were associated with vascular growth induction and (b) 18 were associated with vascular growth inhibition, whose expression was altered by CAD/PVD medium treatment**. The reference numbers are attached as a separate file.

A

| **Positive**  **Vasculogenic Effect** | **Cellular Activity Changes under pseudo-CAD/PVD treatment** | **Gene** | **Direction of Change in Disease Model** |
| --- | --- | --- | --- |
| Angiogenesis | Endothelial Cell Junction | ADTRP (Patel et al. 2018)  NTF3 (Su et al. 2016) | Up |
|  | Angiogenic Rearrangement | CMA1 (Orlowska-Baranowska et al. 2014; Meyer et al. 2017)  MAP3K9 (Fawdar et al. 2013; Nie et al, 2016; Gambino et al. 2017) |  |
|  | Endothelial Migration | Inc-CDH2-1 (Dorrell et al. 2002; Nalla et al. 2011)  GPR15 (Pan et al. 2017) |  |
|  | Angiogenic Sprouting | LIM2 (Dave et al. 2016)  CSPG4 (Shapiro et al. 2017) |  |
|  | Chemokine Response | IL2RG (Bae et al. 2008) |  |
|  | Endothelial Migration | PIK3R5(Woodcock et al. 1997; Lin S et al. 2017)  KRT16P3 (Ahmed et al. 2015)  POSTN (Oka et al. 2007), ANGPTL3 (Camenisch et al. 2002)  ANGPT4 (Kesler et al. 2015)  FMO5 (Zhang et al. 2009; Scott et al. 2017) | Down |
|  | Angiogenic Rearrangement | PTGER3 (Salvado et al. 2013) |  |
|  | Cell-ECM interaction | CELSR1 (Zhan et al. 2016)  EDN3 (Patel et al. 2014)  AQP1 (Nielsen et al. 1993; Shanahan et al. 1999)  PAEP (Pala et al. 2012; Valdez-Morales et al. 2015)  ITGB4, MUC5AC (Lakshmanan et al. 2016) |  |
|  | Chemokine Response | CSF3, CSF2RB (Ohki et al. 2005; Mehta et al. 2015)  IL9 (Singh et al. 2013)  IL17C (Numasaki et al. 2003)  CXCL2, CXCL3(Al-Alwan et al. 2013)  CD160 (Fons et al. 2006; Chabot et al. 2011)  IL33 (Schmitz et al. 2005)  CCL25 (Chen et al. 2012; Deng et al. 2017)  STAT 4 (Sperati et al. 2009; Torpey et al. 2004; Cunnion et al. 2017; Meng et al. 2017)  CAPSL (Santiago et al. 2008)  CD300 C(Raggi et al. 2014) |  |
|  | Angiogenic Sprouting | SCUBE2 (Lin YC et al. 2017)  EGR3 (Liu et al. 2003; Yan et al. 2006) |  |
|  | Circular Canal Development | NR4A3 (Zhao and Bruemmer 2009) |  |
|  | Coronary Vascular Morphogenesis | PDGFR (Battegay et al. 1994)^]^ |  |
|  | Endothelial Cell Junction | CERCAM (Starzyk et al. 2000) |  |
|  | Positive Angiogenic Regulation | PTGIS (da Costa et al. 2008 ; Catalano et al. 2011; Wang et al. 2013)  IGF2 (Chao et al. 2008)  ATP2A3 (Wu et al. 2001) |  |
| Multiple Tissue/Neuronal Vascular Induction | Cell Growth | KRT17 (Kim et al. 2006)  PDE1C, lnc-PDE4D-1 (Lugnier et al. 2006),  lnc-PDE3B-1 (Keravis et al. 2000; Bender et al. 2006)  STAMBPL1 (McCullough et al. 2004; Fraile et al. 2012) | Up |
|  | Cell Migration | PADI6 (Kan et al. 2011) |  |
|  | Cell-Cell Adhesion | NRXN1 (Rissone et al. 2012) |  |
|  | Axon Sprouting | ERBB4 (Liang et al. 2015) |  |
|  | Immune Response | IL5 (Martínez-Varea et al. 2015) | Down |
|  | Insulin Resistance | SHOX (Shaprio et al. 2015) |  |
| Tumorigenic Vascular Genesis | Tumor Growth | GREB1L (Liu et al. 2012; Hnatyszyn et al; 2014)  CDK1 (Warfel et al. 2013)  TYRP1 (Gilot et al. 2017)  FOXR2 (Xu et al. 2017)  PLCD4 (Leung et al. 2004)  SCD5 (Sinner et al. 2012) | Up |
|  | Tumorigenic Vascular Invasion | AKT1S1 (Woodcock et al.1997; Wang et al. 2020)  GSN (Huang et al. 2016)  TGFB2 (Mahmoud et al. 2011; Li et al. 2012)  SERPIND1 (Ikeda et al. 2012)  PVRL4 (Nishiwada et al. 2015)  CASR (Hernández-Bedolla et al. 2015) |  |
|  | Tumor Growth | GALNT14 (Huanna et al. 2015)  GREB1 (Hnatyszyn et al. 2014)  GPM6B (Bilecova-Rabajdova et al. 2014)  SERPINB7, SERPINB10 (Valiente et al. 2014) | Down |
|  | Tumorigenic Vascular Invasion | MKL2 (Li et al. 2012) |  |
|  | Tumorigenic Signal Enhancement | PAK6 (Radu et al. 2014) |  |

B

| Negative  Vasculogenic Effect | Cellular Activity Changes under pseudo-CAD/PVD treatment | Gene | Direction of Change in Disease Model |
| --- | --- | --- | --- |
| Angiogenic Suppression | Inhibit Angiogenesis in Disease | WNT5A (Smadja et al. 2010; Shi et al. 2017)  RORA-AS1 (Sun et al. 2015) | Up |
|  | Negative Angiogenic Regulation | RPF1 (Leung et al. 2004; Shapiro et al. 2015) |  |
|  | Angiogenic Inflammation | GBP5 (Shenoy et al. 2012) |  |
|  | Negative Angiogenic Regulation | FOXP-AS1 (Grundmann et al. 2013)  ISM1 (Xiang et al. 2011) |  |
| Tumorigenic Vascular Suppression | Tumor Suppression | MIA2 (Xu et al. 2011)  EGOT (Jin et al. 2017)  NEURL4 (Cubillos-Rojas et al. 2017)  CDH4 (Xie et al, 2016) | Up |
|  | Tumor Vascular Inhibition | CYP3A5 (Jiang et al. 2015)  TGFB2-AS1(Mahmoud et al. 2011) |  |
|  | Suppression of Epithelial Cell Migration | FST (Li et al. 2014; Seachrist et al. 2017)  CDRT15 (Sun et al. 2016) |  |
|  | Tumor Vascular inhibition | FBLN2 (Icli et al. 2014)  CASC2 (Weng et al. 2017) | Down |
|  | Tumor Suppression | GALNT9 (Pangeni et al. 2015) |  |
| Multiple Tissue/Neuronal Vascular Suppression | Tumor Growth | DKK1 (Smadja et al. 2010; Shi et al. 2017) | Up |

**References**

Ahmed RR, Mahmoud A, Ahmed OM, Metwalli A, Ebaid H (2015) Up-regulation of Hsp72 and keratin16 mediates wound healing in streptozotocin diabetic rats. Biol Res 1;48:54. https://doi.org/10.1186/s40659-015-0044-5

Al-Alwan LA, Chang Y, Mogas A, Halayko AJ, Baglole CJ, Martin JG, Rousseau S, Eidelman DH, Hamid Q (2013) Differential roles of CXCL2 and CXCL3 and their receptors in regulating normal and asthmatic airway smooth muscle cell migration. J Immunol 19:2731-2741. https://doi.org/10.4049/jimmunol.1203421

Bae J, Park D, Lee YS, Jeoung D (2008) Interleukin-2 promotes angiogenesis by activation of Akt and increase of ROS. J Microbiol Biotechnol 18:377-382.

Battegay EJ, Rupp J, Iruela-Arispe L, Sage EH, Pech M (1994) PDGF-BB modulates endothelial proliferation and angiogenesis in vitro via PDGF beta-receptors. J Cell Biol 125:917-928. https://doi.org/10.1083/jcb.125.4.917

Bender AT, Beavo JA (2006) Cyclic nucleotide phosphodiesterases: molecular regulation to clinical use. Pharmacol Rev 58:488-520. https://doi.org/10.1124/pr.58.3.5

Bilecova-Rabajdova M, Urban P, Gregova K, Varga J, Fialkovicová V, Kruzliak P, Marekova M (2014) Breast carcinoma progression and tumour vascular markers related to apoptotic mechanisms. Dis Markers 2014:156034. https://doi.org/10.1155/2014/156034

Camenisch G, Pisabarro MT, Sherman D, Kowalski J, Nagel M, Hass P, Xie MH, Gurney A, Bodary S, Liang XH, Clark K, Beresini M, Ferrara N, Gerber HP (2002) ANGPTL3 stimulates endothelial cell adhesion and migration via integrin alpha vbeta 3 and induces blood vessel formation in vivo. J Biol Chem 277:17281-17290. https://doi.org/10.1074/jbc.M109768200

Catalano RD, Wilson MR, Boddy SC, McKinlay AT, Sales KJ, Jabbour HN (2011) Hypoxia and prostaglandin E receptor 4 signalling pathways synergise to promote endometrial adenocarcinoma cell proliferation and tumour growth. PLoS One 6:e19209. https://doi.org/10.1371/journal.pone.0019209

Chabot S, Jabrane-Ferrat N, Bigot K, Tabiasco J, Provost A, Golzio M, Noman MZ, Giustiniani J, Bellard E, Brayer S, Aguerre-Girr M, Meggetto F, Giuriato S, Malecaze F, Galiacy S, Jaïs JP, Chose O, Kadouche J, Chouaib S, Teissié J, Abitbol M, Bensussan A, Le Bouteiller P (2011) A novel antiangiogenic and vascular normalization therapy targeted against human CD160 receptor. J Exp Med 208:973-986. https://doi.org/10.1084/jem.20100810

Chao W, D'Amore PA (2008) IGF2: epigenetic regulation and role in development and disease. Cytokine Growth Factor Rev 19:111-120. <https://doi.org/10.1016/j.cytogfr.2008.01.005>

Chen HJ, Edwards R, Tucci S, Bu P, Milsom J, Lee S, Edelmann W, Gümüs ZH, Shen X, Lipkin S (2012) Chemokine 25-induced signaling suppresses colon cancer invasion and metastasis. J Clin Invest 122:3184-3196. https://doi.org/10.1172/JCI62110

Cubillos-Rojas M, Schneider T, Bartrons R, Ventura F, Rosa JL (2017) NEURL4 regulates the transcriptional activity of tumor suppressor protein p53 by modulating its oligomerization. Oncotarget 8:61824-61836. <https://doi.org/10.18632/oncotarget.18699>

Cunnion KM, Krishna NK, Pallera HK, Pineros-Fernandez A, Rivera MG, Hair PS, Lassiter BP, Huyck R, Clements MA, Hood AF, Rodeheaver GT, Cottler PS, Nadler JL, Dobrian AD (2017) Complement Activation and STAT4 Expression Are Associated with Early Inflammation in Diabetic Wounds. PLoS One 12:e0170500. https://doi.org/10.1371/journal.pone.0170500

Dave JM, Abbey CA, Duran CL, Seo H, Johnson GA, Bayless KJ (2016) Hic-5 mediates the initiation of endothelial sprouting by regulating a key surface metalloproteinase. J Cell Sci 129:743-756. https://doi.org/10.1242/jcs.170571

Deng X, Tu Z, Xiong M, Tembo K, Zhou L, Liu P, Pan S, Xiong J, Yang X, Leng J, Zhang Q, Xiao R, Zhang Q (2017) Wnt5a and CCL25 promote adult T-cell acute lymphoblastic leukemia cell migration, invasion and metastasis. Oncotarget 8:39033-39047. https://doi.org/10.18632/oncotarget.16559

Dorrell MI, Aguilar E, Friedlander M (2002) Retinal vascular development is mediated by endothelial filopodia, a preexisting astrocytic template and specific R-cadherin adhesion. Invest Ophthalmol Vis Sci 43:3500-3510.

Fawdar S, Trotter EW, Li Y, Stephenson NL, Hanke F, Marusiak AA, Edwards ZC, Ientile S, Waszkowycz B, Miller CJ, Brognard J (2013) Targeted genetic dependency screen facilitates identification of actionable mutations in FGFR4, MAP3K9, and PAK5 in lung cancer. Proc Natl Acad Sci U S A 110:12426-12431. https://doi.org/10.1073/pnas.1305207110

Fons P, Chabot S, Cartwright JE, Lenfant F, L'Faqihi F, Giustiniani J, Herault JP, Gueguen G, Bono F, Savi P, Aguerre-Girr M, Fournel S, Malecaze F, Bensussan A, Plouët J, Le Bouteiller P (2006) Soluble HLA-G1 inhibits angiogenesis through an apoptotic pathway and by direct binding to CD160 receptor expressed by endothelial cells. Blood 108:2608-2615. <https://doi.org/10.1182/blood->2005-12-019919

Fraile JM, Quesada V, Rodríguez D, Freije JM, López-Otín C. Deubiquitinases in cancer: new functions and therapeutic options. Oncogene. 2012 May 10;31(19):2373-88. https://doi.org/10.1038/onc.2011.443

Gambino TJ, Williams SP, Caesar C, Resnick D, Nowell CJ, Farnsworth RH, Achen MG, Stacker SA, Karnezis T (2017) A Three-Dimensional Lymphatic Endothelial Cell Tube Formation Assay to Identify Novel Kinases Involved in Lymphatic Vessel Remodeling. Assay Drug Dev Technol 15:30-43. https://doi.org/10.1089/adt.2016.764

Gilot D, Migault M, Bachelot L, Journé F, Rogiers A, Donnou-Fournet E, Mogha A, Mouchet N, Pinel-Marie ML, Mari B, Montier T, Corre S, Gautron A, Rambow F, El Hajj P, Ben Jouira R, Tartare-Deckert S, Marine JC, Felden B, Ghanem G, Galibert MD (2017) A non-coding function of TYRP1 mRNA promotes melanoma growth. Nat Cell Biol 19:1348-1357. <https://doi.org/10.1038/ncb3623>

Grundmann S, Lindmayer C, Hans FP, Hoefer I, Helbing T, Pasterkamp G, Bode C, de Kleijn D, Moser M (2013) FoxP1 stimulates angiogenesis by repressing the inhibitory guidance protein semaphorin 5B in endothelial cells. PLoS One 8:e70873. https://doi.org/10.1371/journal.pone.0070873

Hernández-Bedolla MA, Carretero-Ortega J, Valadez-Sánchez M, Vázquez-Prado J, Reyes-Cruz G (2015) Chemotactic and proangiogenic role of calcium sensing receptor is linked to secretion of multiple cytokines and growth factors in breast cancer MDA-MB-231 cells. Biochim Biophys Acta 1853:166-182. https://doi.org/10.1016/j.bbamcr.2014.10.011

Hnatyszyn HJ, Liu M, Hilger A, Herbert L, Gomez-Fernandez CR, Jorda M, Thomas D, Rae JM, El-Ashry D, Lippman ME (2010) Correlation of GREB1 mRNA with protein expression in breast cancer: validation of a novel GREB1 monoclonal antibody. Breast Cancer Res Treat 122:371-380. <https://doi.org/10.1007/s10549-009-0584-x>

Huang B, Deng S, Loo SY, Datta A, Yap YL, Yan B, Ooi CH, Dinh TD, Zhuo J, Tochhawng L, Gopinadhan S, Jegadeesan T, Tan P, Salto-Tellez M, Yong WP, Soong R, Yeoh KG, Goh YC, Lobie PE, Yang H, Kumar AP, Maciver SK, So JB, Yap CT (2016) Gelsolin-mediated activation of PI3K/Akt pathway is crucial for hepatocyte growth factor-induced cell scattering in gastric carcinoma. Oncotarget 7:25391-25407. https://doi.org/10.18632/oncotarget.8603

Huanna T, Tao Z, Xiangfei W, Longfei A, Yuanyuan X, Jianhua W, Cuifang Z, Manjing J, Wenjing C, Shaochuan Q, Feifei X, Naikang L, Jinchao Z, Chen W (2015) GALNT14 mediates tumor invasion and migration in breast cancer cell MCF-7. Mol Carcinog 54:1159-1171. https://doi.org/10.1002/mc.22186

Icli B, Dorbala P, Feinberg MW (2014) An emerging role for the miR-26 family in cardiovascular disease. Trends Cardiovasc Med 24:241-248. <https://doi.org/10.1016/j.tcm.2014.06.003>

Ikeda Y, Aihara K, Yoshida S, Iwase T, Tajima S, Izawa-Ishizawa Y, Kihira Y, Ishizawa K, Tomita S, Tsuchiya K, Sata M, Akaike M, Kato S, Matsumoto T, Tamaki T (2012) Heparin cofactor II, a serine protease inhibitor, promotes angiogenesis via activation of the AMP-activated protein kinase-endothelial nitric-oxide synthase signaling pathway. J Biol Chem 287:34256-34263. https://doi.org/10.1074/jbc.M112.353532

Jiang F, Chen L, Yang YC, Wang XM, Wang RY, Li L, Wen W, Chang YX, Chen CY, Tang J, Liu GM, Huang WT, Xu L, Wang HY (2015) CYP3A5 Functions as a Tumor Suppressor in Hepatocellular Carcinoma by Regulating mTORC2/Akt Signaling. Cancer Res 75:1470-1081. https://doi.org/10.1158/0008-5472.CAN-14-1589

Jin L, Quan J, Pan X, He T, Hu J, Li Y, Gui Y, Yang S, Mao X, Chen Y, Lai Y (2017) Identification of lncRNA EGOT as a tumor suppressor in renal cell carcinoma. Mol Med Rep 16:7072-7079. https://doi.org/10.3892/mmr.2017.7470

Kan R, Yurttas P, Kim B, Jin M, Wo L, Lee B, Gosden R, Coonrod SA (2011) Regulation of mouse oocyte microtubule and organelle dynamics by PADI6 and the cytoplasmic lattices. Dev Biol 350:311-322. https://doi.org/10.1016/j.ydbio.2010.11.033

Keravis T, Komas N, Lugnier C (2000) Cyclic nucleotide hydrolysis in bovine aortic endothelial cells in culture: differential regulation in cobblestone and spindle phenotypes. J Vasc Res 37:235-349.https://doi.org/10.1159/000025738

Kesler CT, Pereira ER, Cui CH, Nelson GM, Masuck DJ, Baish JW, Padera TP (2015) Angiopoietin-4 increases permeability of blood vessels and promotes lymphatic dilation. FASEB J 29:3668-3677. https://doi.org/10.1096/fj.14-268920

Kim S, Wong P, Coulombe PA (2006) A keratin cytoskeletal protein regulates protein synthesis and epithelial cell growth. Nature 441:362-365. https://doi.org/10.1038/nature04659

Lakshmanan I, Rachagani S, Hauke R, Krishn SR, Paknikar S, Seshacharyulu P, Karmakar S, Nimmakayala RK, Kaushik G, Johansson SL, Carey GB, Ponnusamy MP, Kaur S, Batra SK, Ganti AK (2016) MUC5AC interactions with integrin β4 enhances the migration of lung cancer cells through FAK signaling. Oncogene 4;35:4112-4121. https://doi.org/10.1038/onc.2015.478

Leung DW, Tompkins C, Brewer J, Ball A, Coon M, Morris V, Waggoner D, Singer JW (2004) Phospholipase C delta-4 overexpression upregulates ErbB1/2 expression, Erk signaling pathway, and proliferation in MCF-7 cells. Mol Cancer 3:15. https://doi.org/10.1186/1476-4598-3-15

Li J, Bowens N, Cheng L, Zhu X, Chen M, Hannenhalli S, Cappola TP, Parmacek MS (2012) Myocardin-like protein 2 regulates TGFβ signaling in embryonic stem cells and the developing vasculature. Development 139:3531-3542. https://doi.org/10.1242/dev.082222

Li X, Liu H, Wang H, Sun L, Ding F, Sun W, Han C, Wang J (2014) Follistatin could promote the proliferation of duck primary myoblasts by activating PI3K/Akt/mTOR signalling. Biosci Rep 34:e00143. https://doi.org/10.1042/BSR20140085

Liang X, Ding Y, Zhang Y, Chai YH, He J, Chiu SM, Gao F, Tse HF, Lian Q (2015) Activation of NRG1-ERBB4 signaling potentiates mesenchymal stem cell-mediated myocardial repairs following myocardial infarction. Cell Death Dis 6:e1765. https://doi.org/10.1038/cddis.2015.91

Lin S, Zhang Q, Shao X, Zhang T, Xue C, Shi S, Zhao D, Lin Y (2017) IGF-1 promotes angiogenesis in endothelial cells/adipose-derived stem cells co-culture system with activation of PI3K/Akt signal pathway. Cell Prolif 50:e12390. https://doi.org/10.1111/cpr.12390

Lin YC, Chao TY, Yeh CT, Roffler SR, Kannagi R, Yang RB (2017) Endothelial SCUBE2 Interacts With VEGFR2 and Regulates VEGF-Induced Angiogenesis. Arterioscler Thromb Vasc Biol 37:144-155. <https://doi.org/10.1161/ATVBAHA.116.308546>

Liu D, Jia H, Holmes DI, Stannard A, Zachary I (2003) Vascular endothelial growth factor-regulated gene expression in endothelial cells: KDR-mediated induction of Egr3 and the related nuclear receptors Nur77, Nurr1, and Nor1. Arterioscler Thromb Vasc Biol 23:2002-2007. <https://doi.org/10.1161/01.ATV.0000098644.03153.6F>

Liu W, Xie S, Chen X, Rao X, Ren H, Hu B, Yin T, Xiang Y, Ren J (2014) Activation of the IL-6/JAK/STAT3 signaling pathway in human middle ear cholesteatoma epithelium. Int J Clin Exp Pathol 7:709-715.

Lugnier C (2006) Cyclic nucleotide phosphodiesterase (PDE) superfamily: a new target for the development of specific therapeutic agents. Pharmacol Ther 109:366-398. https://doi.org/10.1016/j.pharmthera.2005.07.003

Mahmoud M, Upton PD, Arthur HM (2011) Angiogenesis regulation by TGFβ signalling: clues from an inherited vascular disease. Biochem Soc Trans 39:1659-1566. https://doi.org/10.1042/BST20110664

Martínez-Varea A, Pellicer B, Serra V, Hervás-Marín D, Martínez-Romero A, Bellver J, Perales-Marín A, Pellicer A (2015) The Maternal Cytokine and Chemokine Profile of Naturally Conceived Gestations Is Mainly Preserved during In Vitro Fertilization and Egg Donation Pregnancies. J Immunol Res 2015:128616. https://doi.org/10.1155/2015/128616

McCullough J, Clague MJ, Urbé S (2004) AMSH is an endosome-associated ubiquitin isopeptidase. J Cell Biol 166:487-492. https://doi.org/10.1083/jcb.200401141

Mehta HM, Malandra M, Corey SJ (2015) G-CSF and GM-CSF in Neutropenia. J Immunol 195:1341-1349. https://doi.org/10.4049/jimmunol.1500861

Meng ZZ, Liu W, Xia Y, Yin HM, Zhang CY, Su D, Yan LF, Gu AH, Zhou Y (2017) The pro-inflammatory signalling regulator Stat4 promotes vasculogenesis of great vessels derived from endothelial precursors. Nat Commun 8:14640. <https://doi.org/10.1038/ncomms14640>

Meyer N, Woidacki K, Knöfler M, Meinhardt G, Nowak D, Velicky P, Pollheimer J, Zenclussen AC (2017) Chymase-producing cells of the innate immune system are required for decidual vascular remodeling and fetal growth. Sci Rep 7:45106. https://doi.org/10.1038/srep45106

Nalla AK, Estes N, Patel J, Rao JS (2011) N-cadherin mediates angiogenesis by regulating monocyte chemoattractant protein-1 expression via PI3K/Akt signaling in prostate cancer cells. Exp Cell Res 317:2512-2521. https://doi.org/10.1016/j.yexcr.2011.07.024

Nie F, Liu T, Zhong L, Yang X, Liu Y, Xia H, Liu X, Wang X, Liu Z, Zhou L, Mao Z, Zhou Q, Chen T (2016) MicroRNA-148b enhances proliferation and apoptosis in human renal cancer cells via directly targeting MAP3K9. Mol Med Rep13:83-90. https://doi.org/10.3892/mmr.2015.4555

Nielsen S, Smith BL, Christensen EI, Agre P (1993) Distribution of the aquaporin CHIP in secretory and resorptive epithelia and capillary endothelia. Proc Natl Acad Sci U S A 90:7275-7279. https://doi.org/10.1073/pnas.90.15.7275

Nishiwada S, Sho M, Yasuda S, Shimada K, Yamato I, Akahori T, Kinoshita S, Nagai M, Konishi N, Nakajima Y (2015) Nectin-4 expression contributes to tumor proliferation, angiogenesis and patient prognosis in human pancreatic cancer. J Exp Clin Cancer Res 34:30. https://doi.org/10.1186/s13046-015-0144-7

Numasaki M, Fukushi J, Ono M, Narula SK, Zavodny PJ, Kudo T, Robbins PD, Tahara H, Lotze MT (2003)Interleukin-17 promotes angiogenesis and tumor growth. Blood 2003 101:2620-2627. https://doi.org/10.1182/blood-2002-05-1461

Ohki Y, Heissig B, Sato Y, Akiyama H, Zhu Z, Hicklin DJ, Shimada K, Ogawa H, Daida H, Hattori K, Ohsaka A (2005) Granulocyte colony-stimulating factor promotes neovascularization by releasing vascular endothelial growth factor from neutrophils. FASEB J 19:2005-2007. https://doi.org/10.1096/fj.04-3496fje

Oka T, Xu J, Kaiser RA, Melendez J, Hambleton M, Sargent MA, Lorts A, Brunskill EW, Dorn GW 2nd, Conway SJ, Aronow BJ, Robbins J, Molkentin JD (2007) Genetic manipulation of periostin expression reveals a role in cardiac hypertrophy and ventricular remodeling. Circ Res 101:313-321. https://doi.org/10.1161/CIRCRESAHA.107.149047

Orlowska-Baranowska E, Gora J, Baranowski R, Stoklosa P, Gadomska vel Betka L, Pedzich-Placha E, Milkowska M, Koblowska MK, Hryniewiecki T, Gaciong Z, Placha G (2014) Association of the common genetic polymorphisms and haplotypes of the chymase gene with left ventricular mass in male patients with symptomatic aortic stenosis. PLoS One 9:e96306. https://doi.org/10.1371/journal.pone.0096306

Pala A, D'Elia P, Spampinato G, Pittaluga E, Benagiano G (2012) Human amniotic glycodelin actively regulates changes in β-catenin immunoreactivity in cultured human umbilical vein endothelial cells (HUVEC). J Matern Fetal Neonatal Med 25:1514-156. https://doi.org/10.3109/14767058.2011.629258

Pan B, Wang X, Nishioka C, Honda G, Yokoyama A, Zeng L, Xu K, Ikezoe T (2017) G-protein coupled receptor 15 mediates angiogenesis and cytoprotective function of thrombomodulin. Sci Rep 7:692. https://doi.org/10.1038/s41598-017-00781-w

Pangeni RP, Channathodiyil P, Huen DS, Eagles LW, Johal BK, Pasha D, Hadjistephanou N, Nevell O, Davies CL, Adewumi AI, Khanom H, Samra IS, Buzatto VC, Chandrasekaran P, Shinawi T, Dawson TP, Ashton KM, Davis C, Brodbelt AR, Jenkinson MD, Bièche I, Latif F, Darling JL, Warr TJ, Morris MR (2015) The GALNT9, BNC1 and CCDC8 genes are frequently epigenetically dysregulated in breast tumours that metastasis to the brain. Clin Epigenetics 7:57. <https://doi.org/10.1186/s13148->015-0089-x

Patel C, Narayanan SP, Zhang W, Xu Z, Sukumari-Ramesh S, Dhandapani KM, Caldwell RW, Caldwell RB (2014) Activation of the endothelin system mediates pathological angiogenesis during ischemic retinopathy. Am J Pathol 184:3040-3051. https://doi.org/10.1016/j.ajpath.2014.07.012

Patel MM, Behar AR, Silasi R, Regmi G, Sansam CL, Keshari RS, Lupu F, Lupu C (2018) Role of ADTRP (Androgen-Dependent Tissue Factor Pathway Inhibitor Regulating Protein) in Vascular Development and Function. J Am Heart Assoc 7:e010690. https://doi.org/10.1161/JAHA.118.010690

Radu M, Semenova G, Kosoff R, Chernoff J (2014) PAK signalling during the development and progression of cancer. Nat Rev Cancer 14:13-25. https://doi.org/10.1038/nrc3645

Raggi F, Blengio F, Eva A, Pende D, Varesio L, Bosco MC. Identification of CD300a as a new hypoxia-inducible gene and a regulator of CCL20 and VEGF production by human monocytes and macrophages. Innate Immun. 2014 Oct;20(7):721-34. https://doi.org/10.1177/1753425913507095

Rissone A, Foglia E, Sangiorgio L, Cermenati S, Nicoli S, Cimbro S, Beltrame M, Bussolino F, Cotelli F, Arese M (2012) The synaptic proteins β-neurexin and neuroligin synergize with extracellular matrix-binding vascular endothelial growth factor a during zebrafish vascular development. Arterioscler Thromb Vasc Biol 32:1563-1572. https://doi.org/10.1161/ATVBAHA.111.243006

Roberto da Costa RP, Costa AS, Korzekwa AJ, Platek R, Siemieniuch M, Galvão A, Redmer DA, Silva JR, Skarzynski DJ, Ferreira-Dias G (2008) Actions of a nitric oxide donor on prostaglandin production and angiogenic activity in the equine endometrium. Reprod Fertil Dev 20:674-683. https://doi.org/10.1071/rd08015

Salvado MD, Di Gennaro A, Lindbom L, Agerberth B, Haeggström JZ (2013) Cathelicidin LL-37 induces angiogenesis via PGE2-EP3 signaling in endothelial cells, in vivo inhibition by aspirin. Arterioscler Thromb Vasc Biol 33:1965-1972. https://doi.org/10.1161/ATVBAHA.113.301851

Santiago JL, Alizadeh BZ, Martínez A, Espino L, de la Calle H, Fernández-Arquero M, Figueredo MA, de la Concha EG, Roep BO, Koeleman BP, Urcelay E (2008) Study of the association between the CAPSL-IL7R locus and type 1 diabetes. Diabetologia 51:1653-1658. <https://doi.org/10.1007/s00125->008-1070-4

Schmitz J, Owyang A, Oldham E, Song Y, Murphy E, McClanahan TK, Zurawski G, Moshrefi M, Qin J, Li X, Gorman DM, Bazan JF, Kastelein RA (2005) IL-33, an interleukin-1-like cytokine that signals via the IL-1 receptor-related protein ST2 and induces T helper type 2-associated cytokines. Immunity 23:479-490. https://doi.org/10.1016/j.immuni.2005.09.015

Scott F, Gonzalez Malagon SG, O'Brien BA, Fennema D, Veeravalli S, Coveney CR, Phillips IR, Shephard EA (2017) Identification of Flavin-Containing Monooxygenase 5 (FMO5) as a Regulator of Glucose Homeostasis and a Potential Sensor of Gut Bacteria. Drug Metab Dispos 45:982-989. https://doi.org/10.1124/dmd.117.076612

Seachrist DD, Sizemore ST, Johnson E, Abdul-Karim FW, Weber Bonk KL, Keri RA (2017) Follistatin is a metastasis suppressor in a mouse model of HER2-positive breast cancer. Breast Cancer Res 19:66. https://doi.org/10.1186/s13058-017-0857-y

Shanahan CM, Connolly DL, Tyson KL, Cary NR, Osbourn JK, Agre P, Weissberg PL (1999) Aquaporin-1 is expressed by vascular smooth muscle cells and mediates rapid water transport across vascular cell membranes. J Vasc Res 36:353-362. https://doi.org/10.1159/000025674

Shapiro JP, Guzeloglu-Kayisli O, Kayisli UA, Semerci N, Huang SJ, Arlier S, Larsen K, Fadda P, Schatz F, Lockwood CJ (2017) Thrombin impairs human endometrial endothelial angiogenesis; implications for progestin-only contraceptive-induced abnormal uterine bleeding. Contraception 95:592-601. https://doi.org/10.1016/j.contraception.2017.04.001

Shapiro S, Klein GW, Klein ML, Wallach EJ, Fen Y, Godbold JH, Rapaport R (2015) SHOX gene variants: growth hormone/insulin-like growth factor-1 status and response to growth hormone treatment. Horm Res Paediatr 83:26-35. https://doi.org/10.1159/000365507

Shenoy AR, Wellington DA, Kumar P, Kassa H, Booth CJ, Cresswell P, MacMicking JD (2012) GBP5 promotes NLRP3 inflammasome assembly and immunity in mammals. Science 2012 336:481-485. https://doi.org/10.1126/science.1217141

Shi YN, Zhu N, Liu C, Wu HT, Gui Y, Liao DF, Qin L (2017) Wnt5a and its signaling pathway in angiogenesis. Clin Chim Acta 471:263-269. https://doi.org/10.1016/j.cca.2017.06.017

Singh TP, Schön MP, Wallbrecht K, Gruber-Wackernagel A, Wang XJ, Wolf P (2013) Involvement of IL-9 in Th17-associated inflammation and angiogenesis of psoriasis. PLoS One 8:e51752. https://doi.org/10.1371/journal.pone.0051752

Sinner DI, Kim GJ, Henderson GC, Igal RA (2012) StearoylCoA desaturase-5: a novel regulator of neuronal cell proliferation and differentiation. PLoS One 7:e39787. https://doi.org/10.1371/journal.pone.0039787

Smadja DM, d’Audigier C, Weiswald LB, Badoual C, Dangles-Marie V, Mauge L, Evrard S, Laurendeau I, Lallemand F, Germain S, Grelac F, Dizier B, Vidaud M, Bièche I, Gaussem P (2010) The Wnt antagonist Dickkopf-1 increases endothelial progenitor cell angiogenic potential. Arterioscler Thromb Vasc Biol 30:2544-2452. https://doi.org/10.1161/ATVBAHA.110.213751

Sperati CJ, Parekh RS, Berthier-Schaad Y, Jaar BG, Plantinga L, Fink N, Powe NR, Smith MW, Coresh J, Kao WH (2009) Association of single-nucleotide polymorphisms in JAK3, STAT4, and STAT6 with new cardiovascular events in incident dialysis patients. Am J Kidney Dis 53:845-855. https://doi.org/10.1053/j.ajkd.2008.12.025

Starzyk RM, Rosenow C, Frye J, Leismann M, Rodzinski E, Putney S, Tuomanen EI (2000) Cerebral cell adhesion molecule: a novel leukocyte adhesion determinant on blood-brain barrier capillary endothelium. J Infect Dis 181:181-187. https://doi.org/10.1086/315163

Su YW, Chung R, Ruan CS, Chim SM, Kuek V, Dwivedi PP, Hassanshahi M, Chen KM, Xie Y, Chen L, Foster BK, Rosen V, Zhou XF, Xu J, Xian CJ (2016) Neurotrophin-3 Induces BMP-2 and VEGF Activities and Promotes the Bony Repair of Injured Growth Plate Cartilage and Bone in Rats. J Bone Miner Res 31:1258-1274. https://doi.org/10.1002/jbmr.2786

Sun Y, Liu CH, SanGiovanni JP, Evans LP, Tian KT, Zhang B, Stahl A, Pu WT, Kamenecka TM, Solt LA, Chen J (2015) Nuclear receptor RORα regulates pathologic retinal angiogenesis by modulating SOCS3-dependent inflammation. Proc Natl Acad Sci U S A 112:10401-10406. https://doi.org/10.1073/pnas.1504387112

Torpey N, Maher SE, Bothwell AL, Pober JS (2004) Interferon alpha but not interleukin 12 activates STAT4 signaling in human vascular endothelial cells. J Biol Chem 279:26789-26796. https://doi.org/10.1074/jbc.M401517200

Valdez-Morales FJ, Gamboa-Domínguez A, Vital-Reyes VS, Cruz JC, Chimal-Monroy J, Franco-Murillo Y, Cerbón M (2015) Changes in receptivity epithelial cell markers of endometrium after ovarian stimulation treatments: its role during implantation window. Reprod Health 12:45. https://doi.org/10.1186/s12978-015-0034-7

Valiente M, Obenauf AC, Jin X, Chen Q, Zhang XH, Lee DJ, Chaft JE, Kris MG, Huse JT, Brogi E, Massagué J (2014) Serpins promote cancer cell survival and vascular co-option in brain metastasis. Cell 56:1002-1016. https://doi.org/10.1016/j.cell.2014.01.040

Wang C, Chen Y, Chen K, Zhang L (2020) Long Noncoding RNA LINC01134 Promotes Hepatocellular Carcinoma Metastasis via Activating AKT1S1 and NF-κB Signaling. Front Cell Dev Biol 12;8:429.https://doi.org/10.3389/fcell.2020.00429

Wang J, Ikeda R, Che XF, Ooyama A, Yamamoto M, Furukawa T, Hasui K, Zheng CL, Tajitsu Y, Oka T, Tabata S, Nishizawa Y, Eizuru Y, Akiyama S (2013) VEGF expression is augmented by hypoxia‑induced PGIS in human fibroblasts. Int J Oncol 43:746-754. https://doi.org/10.3892/ijo.2013.1994

Warfel NA, Dolloff NG, Dicker DT, Malysz J, El-Deiry WS (2013) CDK1 stabilizes HIF-1α via direct phosphorylation of Ser668 to promote tumor growth. Cell Cycle 12:3689-3701. https://doi.org/10.4161/cc.26930

Wu KD, Bungard D, Lytton J (2001) Regulation of SERCA Ca2+ pump expression by cytoplasmic Ca2+ in vascular smooth muscle cells. Am J Physiol Cell Physiol 280:C843-C851. https://doi.org/10.1152/ajpcell.2001.280.4.C843

Xiang W, Ke Z, Zhang Y, Cheng GH, Irwan ID, Sulochana KN, Potturi P, Wang Z, Yang H, Wang J, Zhuo L, Kini RM, Ge R (2011) Isthmin is a novel secreted angiogenesis inhibitor that inhibits tumour growth in mice. J Cell Mol Med 15:359-374. https://doi.org/10.1111/j.1582-4934.2009.00961.x

Xie J, Feng Y, Lin T, Huang XY, Gan RH, Zhao Y, Su BH, Ding LC, She L, Chen J, Lin LS, Lin X, Zheng DL, Lu YG (2016) CDH4 suppresses the progression of salivary adenoid cystic carcinoma via E-cadherin co-expression. Oncotarget 7:82961-82971. https://doi.org/10.18632/oncotarget.12821

Xu W, Chang J, Liu G, Du X, Li X (2017) Knockdown of FOXR2 suppresses the tumorigenesis, growth and metastasis of prostate cancer. Biomed Pharmacother 87:471-475. https://doi.org/10.1016/j.biopha.2016.12.120

Xu Y, Yang Y, Cai Y, Liu F, Liu Y, Zhu Y, Wu J (2011) The X protein of hepatitis B virus activates hepatoma cell proliferation through repressing melanoma inhibitory activity 2 gene. Biochem Biophys Res Commun 416(3-4):379-84. https://doi.org/10.1016/j.bbrc.2011.11.046

Yan SF, Harja E, Andrassy M, Fujita T, Schmidt AM (2006) Protein kinase C beta/early growth response-1 pathway: a key player in ischemia, atherosclerosis, and restenosis. J Am Coll Cardiol 48:A47-A55. https://doi.org/10.1016/j.jacc.2006.05.063

Zhan YH, Luo QC, Zhang XR, Xiao NA, Lu CX, Yue C, Wang N, Ma QL (2016) CELSR1 Is a Positive Regulator of Endothelial Cell Migration and Angiogenesis. Biochemistry (Mosc) 2016 81:591-599. https://doi.org/10.1134/S0006297916060055

Zhao Y, Bruemmer D (2009) NR4A Orphan Nuclear Receptors in Cardiovascular Biology. Drug Discov Today Dis Mech 6:e43-e48. https://doi.org/10.1016/j.ddmec.2009.06.001

Zhang J, Chaluvadi MR, Reddy R, Motika MS, Richardson TA, Cashman JR, Morgan ET (2009) Hepatic flavin-containing monooxygenase gene regulation in different mouse inflammation models. Drug Metab Dispos 37:462-468. Https://doi.org/10.1124/dmd.108.025338
