# Supplementary material for: A simplified in vitro disease-mimicking culture system can determine the angiogenic effect of medicines on vascular diseases
Source: Cytotechnology. 2025 Mar 7;77(2):75. doi: 10.1007/s10616-025-00736-4 (PMC11889311; doi:10.1007/s10616-025-00736-4)
Supplement: Supplementary file 8 — Supplementary file8 (DOCX 1193 KB) [file 10616_2025_736_MOESM8_ESM.docx]

**Supplementary Information**

**A simplified *in vitro* disease-mimicking culture system can determine the angiogenic effect of medicines on vascular diseases**

SongHo Moon^1^, Yuzuru Ito^1,2,3*^

^1^Faculty of Life and Environmental Sciences, University of Tsukuba, Tsukuba, Ibaraki, Japan

^2^Life Science Development Department, CHIYODA Corporation, Yokohama, Kanagawa, Japan

^3^National Institute of Advanced Industrial Science and Technology (AIST), Tsukuba, Ibaraki, Japan

*Corresponding author

Yuzuru Ito

ORCID ID: 0000-0001-7923-865X

Email: [ito.yuzuru.fe@u.tsukuba.ac.jp](mailto:ito.yuzuru.fe@u.tsukuba.ac.jp)

**
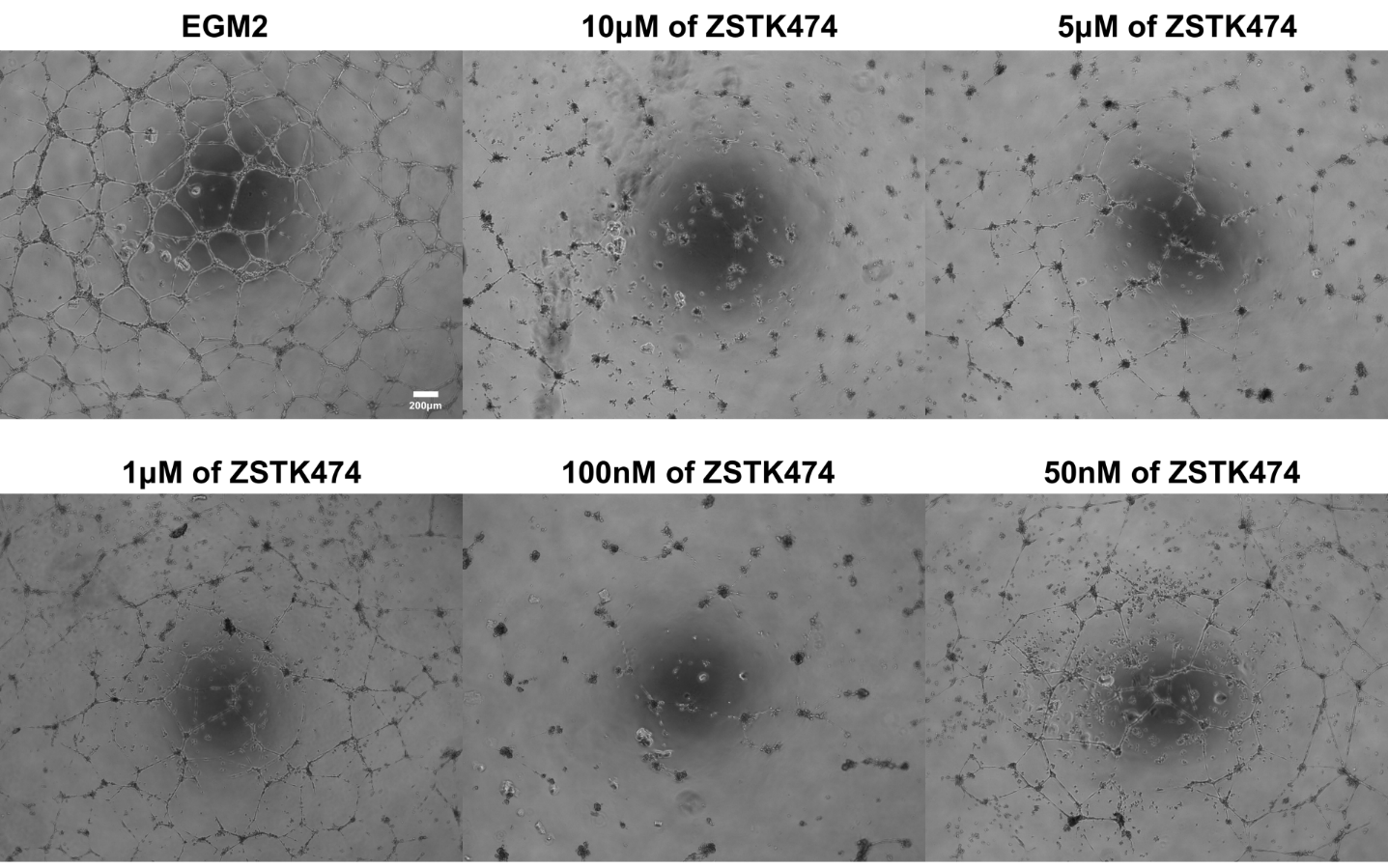
**

**Online Resource 8 Effects of treatment with various concentrations of a PI3K/AKT inhibitor during the tube formation assay.** EGM2: control human umbilical vein endothelial cells (HUVECs); ZSTK474: HUVECs treated with the indicated concentrations of the phosphatidylinositol 3-kinase/protein kinase B (PI3K/Akt) inhibitor ZSTK474 during the tube formation assay
